# Oil Displacement in Calcite-Coated Microfluidic Chips via Waterflooding at Elevated Temperatures and Long Times

**DOI:** 10.3390/mi13081316

**Published:** 2022-08-14

**Authors:** Duy Le-Anh, Ashit Rao, Amy Z. Stetten, Subhash C. Ayirala, Mohammed B. Alotaibi, Michel H. G. Duits, Han Gardeniers, Ali A. AlYousef, Frieder Mugele

**Affiliations:** 1 Physics of Complex Fluids, MESA+ Institute, Faculty of Science and Technology, University of Twente, P.O. Box 217, 7500 AE Enschede, The Netherlands; 2 The Exploration and Petroleum Engineering Center-Advanced Research Center (EXPEC ARC), Saudi Aramco, Dhahran 34465, Saudi Arabia; 3 Mesoscale Chemical Systems Groups, MESA+ Institute, Faculty of Science and Technology, University of Twente, P.O. Box 217, 7500 AE Enschede, The Netherlands

**Keywords:** microfluidics, oil recovery, waterflooding, calcite, pore visualization, osmotic swelling

## Abstract

In microfluidic studies of improved oil recovery, mostly pore networks with uniform depth and surface chemistry are used. To better mimic the multiple porosity length scales and surface heterogeneity of carbonate reservoirs, we coated a 2.5D glass microchannel with calcite particles. After aging with formation water and crude oil (CRO), high-salinity Water (HSW) was flooded at varying temperatures and durations. Time-resolved microscopy revealed the CRO displacements. Precise quantification of residual oil presented some challenges due to calcite-induced optical heterogeneity and brine–oil coexistence at (sub)micron length scales. Both issues were addressed using pixel-wise intensity calibration. During waterflooding, most of the ultimately produced oil gets liberated within the first pore volume (similar to glass micromodels). Increasing temperature from 22 °C to 60 °C and 90 °C produced some more oil. Waterflooding initiated directly at 90 °C produced significantly more oil than at 22 °C. Continuing HSW exposure at 90 °C for 8 days does not release additional oil; although, a spectacular growth of aqueous droplets is observed. The effect of calcite particles on CRO retention is weak on flat surfaces, where the coverage is ~20%. The calcite-rich pore edges retain significantly more oil suggesting that, in our micromodel wall roughness is a stronger determinant for oil retention than surface chemistry.

## 1. Introduction

In the past decade, the microfluidics platform emerged as a promising complementary tool for studying processes related to oil recovery, at low cost and high speed. Compared to coreflooding experiments, studies of improved oil recovery (IOR) through microfluidic waterflooding offer the advantage of real-time visualization, while compared to millifluidic models, microfluidics offers micron length scales which are pertinent to many oil-bearing rocks. Recently, X-ray micro-computer-tomography has also become capable of addressing micron length scales, but this technique requires contrast agents. While the development of the so-called IOR-on-a-Chip [1,2,3,4] started with glass/silicon micromodels studied at room temperature [5,6], and alkane [7,8,9] or model oils [10,11], significant improvements are currently being made along several lines.

Temperature control of the pore space is crucial for mimicking the elevated temperatures encountered in many subterranean reservoirs. Depending on the oil field and on the reservoir depth, the temperature varies typically from 50 to ~120 °C, along with enhanced pressures. Recent microfluidic studies have mimicked IOR at elevated temperatures up to 100 °C and ambient pressure [12,13] while very recently the combination of high temperature and high pressure was also addressed [14,15,16]. The waterflooding temperature was found to have significant effects on the amount of produced oil, expressed via the residual oil saturation (ROS) and, in some cases, a relation with the temperature-dependent underlying processes could be elucidated [12,13,16].

The use of crude oils (CROs) rather than model fluids without the surface-active asphaltenes and resins is another major step in better mimicking the oil recovery process. Adsorption and desorption of these amphiphilic compounds on the rock–liquid and liquid–liquid interfaces (where brine and CRO are the two liquids) are of key importance to the wettability alterations that are often required in waterflooding strategies [17,18,19,20,21]. In improved oil recovery, a simple change in ionic composition of the flooding brine can change the presence, structure and composition of interfacial layers [22]. The use of real crude oils also allows to mimic the geochemical aging which takes place in reservoirs; particularly in combination with elevated temperature, such aging can strongly change the chemical nature of the pore surfaces [23,24,25].

The chemical composition of the rock surface represents another aspect that is being considered when developing more realistic microfluidic CRO/Brine/Rock systems. Glass micromodels provide silicon dioxide surfaces which somewhat resemble sandstone rock through the presence of surface silanol groups. Some present-day sandstone reservoirs that are very low in organic surface content [5,6] can display these silanol groups, but in many reservoirs the rock is covered by adsorbed species. Improvements in the mimicking of sandstone reservoirs are made by adsorbing (model) clay particles [25,26,27,28].

Calcite reservoirs are more difficult to mimic via microfluidics, which could explain why only few microfluidic studies with this scope have appeared until now [29,30,31,32]. Some calcite-coated microfluidic chips were made by functionalizing a glass micromodel with calcite nanoparticles. While this approach offers the calcite surface chemistry, the coating density and spatial distribution of the particles appear as new parameters that could play a role in the outcome of waterflooding experiments. In brines that can dissolve the calcite, these parameters can even become time dependent. These aspects related to the discrete nature of the calcite particles have hardly been addressed up until now. An alternative way of making micromodels for IOR in calcite reservoirs is to etch the pore space in a macroscopic calcite slab [33,34,35]. This approach offers a sustained fluid–calcite contact, even if the calcite is dissolving. However, the etching and sealing of such micromodels appears more challenging than for glass-based chips, which could explain why mostly simple pore geometries are used in these studies.

In real rock reservoirs, the geometry of the pore network also plays an important role. Reservoir rocks often show multiple pore length scales or ‘dual porosity’. This aspect can be taken into account either by making packed beds of micron-sized particles (e.g., [36]) or by etching a multiscale porous network. The former option is simple, but often comes with the drawback of a limited optical access (only a few µm deep into the porous medium); while with the latter option, an additional chemical functionalization can still be required after the etching, as discussed above.

Considering these various ongoing developments in ‘microfluidics for IOR’, a clear trend towards a better mimicking of the IOR process is observed, but there is still a lack of microfluidic studies that combine the specific conditions of IOR (elevated temperature, crude oil, aging, surface chemistry and pore heterogeneity). With the present work we want to make a further step in mimicking reservoir conditions, while focusing on calcite reservoirs.

In a previous study [37], we used a glass microchannel to mimic several aspects of IOR from sandstone reservoirs: (i) aging in formation brine and crude oil at elevated temperature, (ii) waterflooding at different temperatures, (iii) use of a dual porosity or ‘2.5D’ network. The principal extensions in the current work are: (a) to partially coat the chip with calcite and (b) to address longer timescales. With aspect (a), we want to study the effect of the present calcite particles on the dynamic brine and CRO distributions during waterflooding, after controlling and characterizing the calcite coating density.

Aspect (b) is not strictly related to the development of ‘realistic’ microfluidics chips for IOR, but addresses an apparent controversy in the literature about the time duration that is needed for flooding brine, to change the (dis)assembly of interfacial layers. While several studies reported waterflooding experiments that lasted ~1 h and showed no further changes after [5,6], other works showed that, in some cases, several days are needed for the brine to effect a wettability alteration [27,38].

To perform these experiments, we adapt methods from the literature to coat a 2.5D glass chip with calcite nanoparticles, and subsequently grow them to the micron scale. Optical microscopy at different magnifications is used to study the evolution of the spatial brine and CRO distributions for the entire pore space and individual pores. Additional image analysis tools are developed to take into account the optical heterogeneities introduced by the calcite particles. Our microfluidic platform and associated analytical methods provide the opportunity to assess fluid displacement and wettability alteration phenomenology close to the actual waterflooding conditions applied to oil reservoirs.

## 2. Materials and Methods

### 2.1. Chemicals

Deionized (DI) water with a conductivity of 18.2 MΩ cm^−1^ was obtained from a Millipore Synergy instrument. All salts (NaCl, CaCl_2_, MgSO_4_, NaHCO_3_), organic solvents (acetic acid, ethanol, isopropanol (IPA), toluene, xylene), 1 M NaOH solution and silane coupling agent (3-trimethoxysilyl-propyl methacrylate, TPM) were purchased from Sigma Aldrich and used as received.

Artificial formation water (FW), high-salinity water (HSW) with ion compositions given in Table S1 (Supporting Information) were prepared by dissolving the salts overnight under stirring at room temperature, followed by filtering through a 0.45 µm polyethersulfone (PES) membrane. Crude oil (CRO) was obtained from a carbonate reservoir with the absence of the dissolved gases and volatile components. The results of a chemical characterization by Saybolt Nederland B.V. are given in Table S2. Temperature-dependent interfacial tensions (IFTs) between CRO and brine, measured using a DataPhysics OCA 20 L apparatus and inverted pendant drops, are shown in Table S3.

To facilitate the coating of the inner chip surfaces with calcite particles (see Section 2.2), a ‘precursor liquid’ was used: a photo-curable mixture of 1,6-hexanediol diacrylate, Mn 250 (HDDA) (technical grade, 80%, Sigma Aldrich, Amsterdam, The Netherlands), photo-initiator 2 wt% phenylbis(2,4,6-trimethyl benzoyl) phosphine oxide (97%, powder, Sigma Aldrich, Amsterdam, The Netherlands), and photo-absorber 0.05 wt% Sudan I (CAS: 842-07-9, Acros Organics, Geel, Belgium). After making a 20 wt% solution of the precursor liquid in IPA, calcite nanoparticles (diameters: 15–40 nm) purchased from SkySpring Nanomaterials were added up to 1.0 wt%. This ‘seed solution’ was sonicated for 3 h to disperse the particles. These seed crystals were chosen because of their polymorphic stability and well-known growth mechanisms [29,39]. To achieve outgrowth of the calcite particles, a Ca^2+^ and CO_3_^2−^ rich ‘ionic solution’ was prepared by carefully mixing equal amounts of 40 mM NaHCO_3_ and 1.6 mM CaCl_2_ solutions in DI water.

### 2.2. Calcite Coating on 2.5D Microchannel

The etch masks for the 2.5D microchannel pore space (see Figure S1) were based on a real rock structure. Dual depth/porosity is encountered in oil reservoirs [40,41] and is represented in our microchannel by ‘deep’ (27 µm) and ‘shallow’ (12 µm) pores, where the deep pores have a typical width of 200 µm, while it is 55 µm for the shallow pores. The arrangement of the pores is such that the fluid cannot traverse the chip without encountering both deep and shallow pores: see Figure S2 and ref. [42].

The fabrication of the calcite-coated 2.5D microchannel includes three main procedures: (i) fabrication of the glass microchannel; (ii) calcite nanoparticle (CalNP) seeding along the inner surfaces; and (iii) in situ outgrowth of the surface-adhering CalNPs. The design and fabrication of the 2.5D glass microchannel are described in detail elsewhere [37]. The calcite coating step was adapted from a previous study [43].

**Figure 1:**
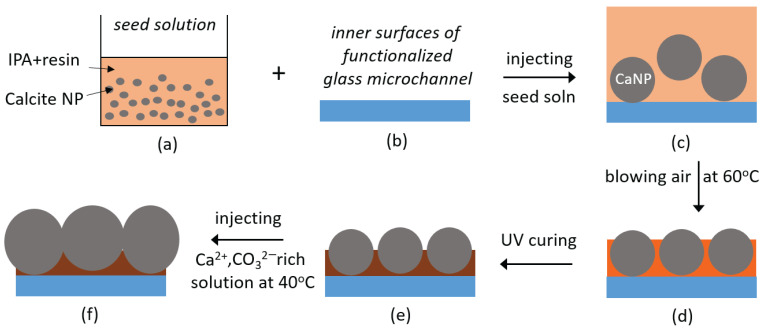
Schematic illustration of the process to coat the inner surfaces of microchannel with micron-sized calcite particles. (**a**–**c**) a seed solution, containing calcite nanoparticles and photocurable polymer is injected into the functionalized 2.5D glass microchannel. (**c**,**d**) blowing with hot air removes the solvent and promotes surface-binding of the CalNPs. Steps (**a**–**d**) are repeated three times. (**d**,**e**) exposing to UV light solidifies the matrix and enhances the CalNP immobilization. (**e**,**f**) adding a solution rich in Ca^2+^ and CO_3_^2−^ leads to outgrowth of the exposed CalNP surfaces. This step is repeated four times. Drawings are not to scale, and calcite crystals are simplistically represented as spheres.

A schematic of the calcite coating process is shown in Figure 1. Firstly, the 2.5D glass microchannel is injected with 1 M aqueous NaOH solution for 1 h, followed by rinsing with DI water. Then, the microchannel is functionalized with 4% *v/v* TPM and 1% *v/v* acetic acid in ethanol by injecting solution at 5.0 μL/min at room temperature for 2 h, to introduce acrylate groups on the inner surface. After a thorough rinsing with ethanol and then DI water, the channel is cured at 80 °C for 30 min. This protocol creates the ‘functionalized glass microchannel’ shown in Figure 1b.

Secondly, to generate a thin layer of CalNPs on the inner surface of the functionalized glass micromodel, the seed solution is injected at a rate of 5.0 μL/min for 10 min. The microchannel is purged with air and cured at 60 °C for 5 min and cooled to room temperature (RT). Three such cycles are performed to deposit a thin layer of seed particles. An example of the microchannel after three cycles is shown in Figure S3. The microchannel is then exposed to UV light for 1 h to cure the monomers in the surface layer, and thereby attach the calcite NPs more firmly. Related to the wetting properties, calcite particles will be sticking out of the cured layer [43], thereby offering surface areas of bare calcite.

Finally, to grow the adhering CalNPs into calcite crystals, the Ca^2+^ and CO_3_^2−^ containing ionic solution is injected at 5.0 μL/min into the microchannel. This flow is strong enough to remove weakly adhering CalNPs while it is still laminar (Reynolds number ~0.1). To avoid the trapping of air bubbles, we initially inject a 50/50 mixture of ethanol and ionic solution [43,44]. Next, pure ionic solution is pumped through the channel for 30 min (~250 Pore Volumes) at 40 °C to achieve growth of the surface-bound calcite. This step is performed four times, each with a freshly made ionic solution, after which the chip is purged with air and dried in an oven at 60 °C. These repeated steps lead to calcite crystals that are large enough to visualize with optical microscopy, while the calcite coverage is also increased. Figure S4 shows a section of the microchannel in which the CalNPs grow with time.

**Figure 2:**
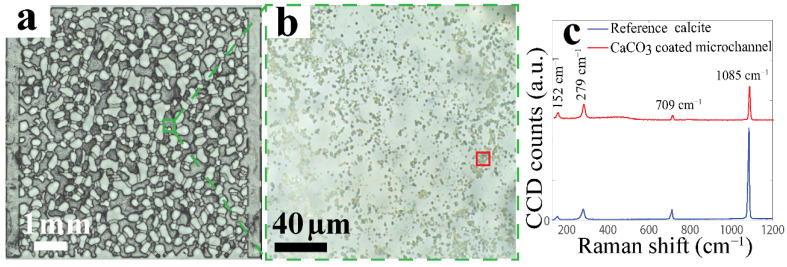
(**a**,**b**) Optical images of the calcite coated 2.5D glass microchannel: (**a**) Entire microchannel and (**b**) at randomly chosen deep pore at 50× magnification. (**c**) Representative Raman spectra of the calcite particles grown on the glass surface (red) and reference calcite (blue).

While optimizing the method, we initially used a CalNP concentration of 1.0 wt%. Figure 2 shows the obtained 2.5D microchannel coated with CaCO_3_ particles along with a Raman spectrum. These data were collected with a Witec 300R Raman microscope with a 50× objective. Raman spectra of the grown particles show Raman peaks at 1084 cm^−1^ (symmetric stretching CO_3_, ν_1_), 711 cm^−1^ (in-phase bending CO_3_, ν_4_), 282 and 157 cm^−1^ (calcite lattice modes), in agreement with the reference calcite. From the optical transmission image, we estimate the total calcite coverage on the horizontal surfaces to be ~70%, with the remarks that (i) we cannot distinguish between the upper and lower surfaces, and (ii) small (<1 μm) calcite crystals cannot be detected. At this high coverage, blocking of the flow occurs in some pores. Therefore, we lowered the CalNP concentration in the seeding solution to 0.4 wt% in our protocol. This leads to lower coverage, especially in the flat pore centers; the pore edges remain densely populated. Even when imaging the entire pore space as we did, a precise quantification of the calcite coverage is not possible with optical or Raman microscopy. This also means that we cannot exclude small differences in calcite coverage even for microchannels that were prepared with the same protocol. Finally, we performed a stability test on the calcite coating by injecting FW at 30 μL/min (much higher than the 0.2 μL/min during waterflooding) for 1 h. The majority (more than 90%) of the calcite particles remained stably attached during this mechanical treatment. The typical time to prepare and characterize a calcite coated chip is one week.

### 2.3. Aging and Waterflooding Protocol

The aging and waterflooding experiments were carried out with the micromodels embedded in a home-made thermostated holder, placed on the stage of a Zeiss Axioskop upright microscope equipped with a Basler (type a2A5328-15ucBAS) 5328 × 4608 pixel color CCD camera (see Supplementay Figure S5 for an overview and ref. [37] for more details). The micromodel was kept on the microscope stage during the entire experiment, i.e., both the aging and the waterflooding. Use of a 2× objective allowed to capture the entire (~7 mm × 7 mm) pore space in a single image (~1.6 μm/pixel). A 10× objective was used to acquire high-resolution still images (~0.32 μm/pixel) of selected pores.

To mimic the in situ reservoir conditions before waterflooding [37], we aged the calcite-coated microchannel with formation water, and subsequently with crude oil. First, ethanol was used to wet the pores and minimize the risk of trapping air bubbles. After that, 20 pore volumes (PVs) of FW were pumped through the chip to ensure complete removal of the ethanol. Formation water and crude oil were successively injected at RT, at a rate of 0.2 μL/min. After heating to 95 °C (at a rate of 5 °C/min), the chip was kept at 95 °C for a given dwell time and then cooled to RT again. The dwell time was 1.5 h for FW and 22 h for CRO, to take into account that CRO molecules need more time to adsorb than ions from the FW. For the CRO, injection was sustained during the entire 22 h to ensure a uniform filling of all pores with CRO. A close-up image of the microchannel, taken during the aging with FW is shown in Figure S6.

Waterflooding was performed with high-salinity water (HSW). In the first experiment, injection of HSW was carried out at 0.2 µL/min for 3.5 h (corresponding to 70 PV) at 22 °C, 3 h at 60 °C and 2 days at 90 °C. These temperatures were chosen from practical considerations: the 68 °C wide range allows to examine temperature dependence, while the 90 °C presents a typical elevated temperature that can still be reached without the need to suppress water boiling. The temperature was increased at a rate of 5 °C/min during the transitions. Passing through the temperatures, the capillary number changes from ∼6 × 10^−6^ to ∼8 × 10^−7^ (see Table S3 for the viscosities and interfacial tensions). The HSW injection was then stopped, and the chip was equilibrated at 90 °C for 8 days, after which the HSW injection was resumed to check for further oil recovery. The second experiment was performed directly at 90 °C, injecting HSW for 24 h. In both experiments, a 2× objective was used to capture the entire pore space as a function of time, while a 10× objective was used intermittently, to acquire zoom-in images at the end of each stage.

### 2.4. Image Analysis

Measurement of the amounts of brine and crude oil in the pore space was performed by calculating the residual oil saturation (ROS) pixelwise (using the 2× objective). Firstly, the deep pores, shallow pores and glass structures were separated from each other by applying MATLAB’s color thresholder to an image of a CRO-filled microchannel: for this liquid, the optical path lengths of the different pore depths (0, 12, and 27 μm) give the strongest color and intensity differences. This thresholding produces overlay masks, allowing to visualize and analyze one type of pore at a time. These masks were used to analyze all microscope images that were collected in a time-series at the same magnification. To correct for slight shifts (μm scale) or rotations (order of 0.1 degree) of the chip with respect to the camera (e.g., after changing the temperature), we used MATLAB’s Registry Estimator toolbox to align the images to the masks. Next, the masked RGB color images were converted into grayscale ones, to enable ROS quantification based on a single intensity scale. After this conversion, we calculated the oil saturation for the deep pores, which represent more than 90% of the total pore volume.

**Figure 3:**
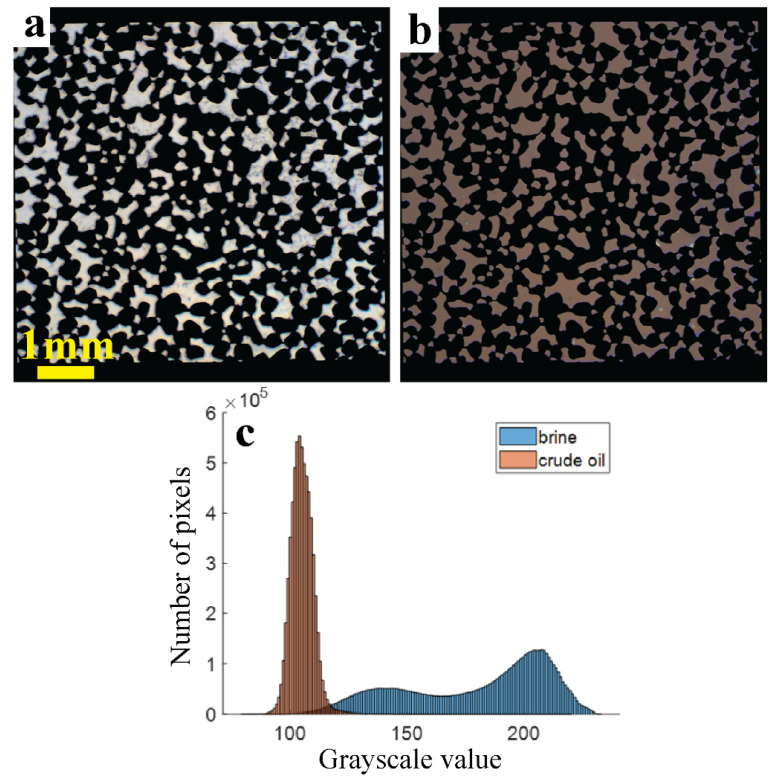
Masked color images of the deep pores filled with (**a**) formation water and (**b**) crude oil at 22 °C, and (**c**) the grayscale intensity histograms derived from them. Pixels corresponding to the areas that are filtered out by the mask have a gray value of zero and are not shown.

Figure 3a,b show masked images of the deep pores filled with FW and CRO (these images were taken in the aging stage). The histograms of the corresponding grayscale values are shown in Figure 3c. Each (X,Y) pixel that contributes to the histogram, corresponds to a (X,Y,Z) volume element inside the pore space. The CRO-filled pores show a narrow distribution of grayscales (centered around 110), while a much broader distribution is seen (two peaks around 140 and 210) for the FW-filled pores. The peak at gray value ~140 for the brine is largely due to the calcite particles. The good separation between histograms means that, as long as the small volume elements contain either pure brine or pure oil, the total amounts of brine/oil can be quantified via a simple intensity thresholding (using a gray value ~115).

The residual oil saturation, defined as the fraction of the pore volume that remains filled with CRO, is used to describe the efficacy of waterflooding. We assume that the deep pores have a uniform depth of 27 μm. The total ROS is then calculated by simply averaging over all pixels that are ‘filtered in’ by the overlay mask. We use two methods to calculate the local ROS: (i) binarization and (ii) interpolation. In the commonly used binarization method [26,31,35,38,40], each pixel is classified as either brine or CRO, depending on the grayscale intensity lying above or below a chosen threshold value. As we will see, however, this method is not suited to resolve non-binary distributions of brine and CRO, and neither to distinguish between calcite and non-calcite.

**Figure 4:**
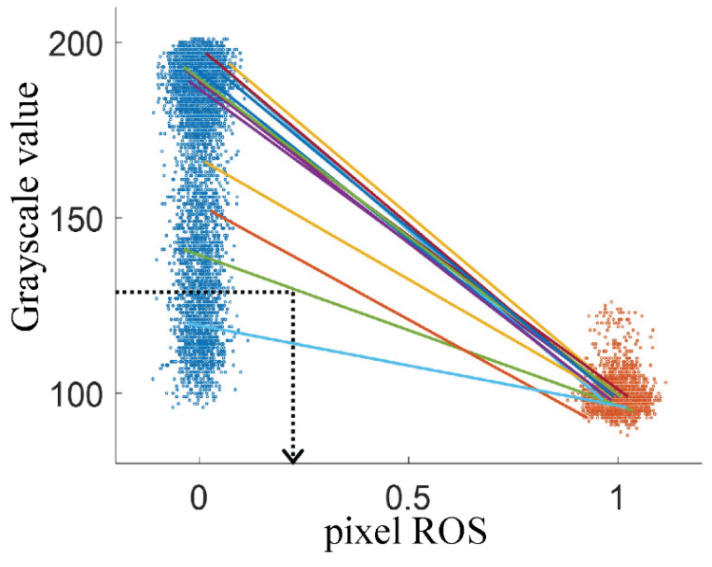
Interpolation method to measure the oil fraction for each (X,Y) pixel. The point clusters correspond to an arbitrary selection of the same 2500 pixels for the chip filled with pure brine (blue) and pure oil (brown). Solid lines connect the gray values of brine and CRO for the same (randomly selected) pixel. For illustrative purposes only, the corresponding oil fractions of 0 and 1 were supplemented with a small random noise. The dotted lines illustrate the interpolation for a single pixel: An observed grayscale value of ~130 during waterflooding translates into a ROS of ~0.22 for that pixel.

To address these shortcomings, we also implemented an interpolation method for calculating ROS. This method is also pixel-based but uses the grayscale intensities as measured in the FW-filled (ROS = 0) and CRO-filled (ROS = 1) chip to define a unique calibration line for each pixel. Figure 4 illustrates the working principle: each line (only a few are drawn) connecting a point in the FW-cloud to a point in the CRO-cloud, corresponds to a specific pixel. This line is then used for calculating the ROS value of that pixel (which changes during waterflooding), via linear interpolation. The grayscale values of FW and HSW are here considered to be the same. This calculation method avoids the error that is introduced by thresholding all pixels with the same grayscale value, and returns fractional ROS values per (X,Y) pixel. As a remark, Figure 3 also implies that with optical microscopy, the presence or absence of calcite particles cannot be confirmed for the pixels that are covered by CRO.

Both methods rely on a constant illumination intensity. The graph shown in Figure S7 illustrates that the grayscale intensities measured on the glass areas (‘pore depth’: 0 µm) were indeed very close to constant. The interpolation method also assumes that slight misalignments between the three used images (in spite of the image registration) do not affect the corresponding pixel intensities. We verified that the image registration is accurate within roughly three pixels (about 5 μm at 2× magnification); this is much smaller than the typical range over which the brine and oil distributions change.

## 3. Results

### 3.1. Residual Oil Saturation

**Figure 5:**
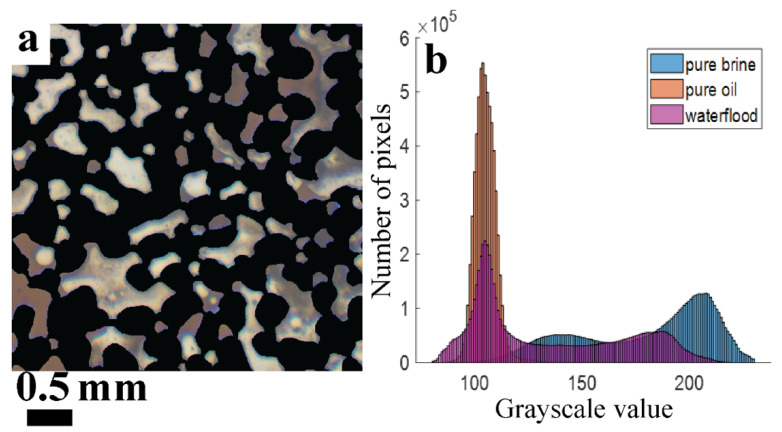
(**a**) Zoomed image of some deep pores after the first 30 min of flooding with HSW at 22 °C. While some pores appear to be filled with pure brine or pure crude oil, there are also pores that show intermediate colors. (**b**) Corresponding grayscale histogram (magenta) compared to those for brine (blue) and oil (brown).

Under all explored waterflooding conditions, it turned out that the fluids in the deep pores showed colors that were not observed during the aging of the chip with pure brine and pure CRO. The intermediate brown shades shown by many (X,Y) pixels in Figure 5a suggest the coexistence of separated brine and CRO layers in the same (X,Y,Z) voxel. The corresponding grayscale histograms in Figure 5b confirm this picture: the histogram measured during waterflooding cannot be decomposed into the histograms for the pure brine and CRO. This means that the usual classification of (X,Y) pixels as being either brine or CRO filled based on intensity thresholding introduces a ‘rounding error’ at the pixel level, while also giving a misleading physical picture. It is not obvious where precisely to choose the grayscale threshold: considering only the waterflooding histogram (magenta), it could be around ~142 (about halfway between the two peaks). However, looking at the reference histogram for brine (blue), it is clear that some pixels, if they were filled with brine during the waterflooding, would be incorrectly counted as CRO. This type of error is obtained with any chosen threshold value around 142.

Our alternative method that calculates a fractional ROS value at the pixel level (see Section 2.4) addresses this error. However, this method is also not fully quantitative. As shown in Figure 5b, there is a small fraction of pixels for which the grayscale intensity is lower than that of pure CRO. These ‘darker structures’ are ascribed to light scattering (see discussion section), and give rise to calculated ROS-values that slightly exceed 1.0 (using linear calibration curves shown in Figure 4 now for extrapolation). Since neither image analysis method can produce an entirely correct ROS number, while the sources of error are different, we will present results for both, denoting the calculation methods as ‘binarization’ and ‘interpolation’. For the shallow pores, the ROS quantification is more challenging because already the grayscale histograms of pure brine and CRO show less well-defined peaks; see Figure S8. Since the shallow pores also occupy less than 10% of the total volume, we exclude them from the analysis.

### 3.2. Effect of Temperature and Time

Previous work on glass micromodels with the same pore network [37] showed two clear trends: (i) the residual oil saturations obtained after flooding many pore volumes of brine were strongly determined by the displacements in the first PV, and (ii) a higher flooding temperature produced a lower ROS. Here, we examine these trends for the calcite-coated chip, and extend the duration of the brine exposure to several days to study the possible influence of slow processes on ROS.

#### 3.2.1. Short-Time Effect for Varying Temperature (History)

**Figure 6:**
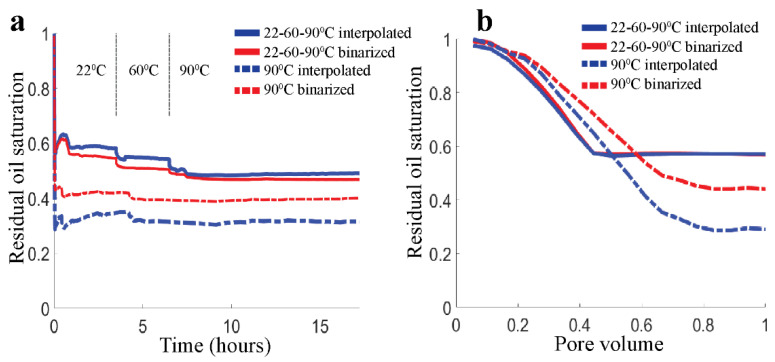
(**a**) Residual Oil Saturation as a function of time for 2 waterflooding experiments. Brine is injected continuously to recover CRO. (i) 3 subsequent regimes (separated by vertical dash-dotted lines) with T = 22, 60 and 90 °C (solid curves), (ii) T = 90 °C (dash-dotted curves). In both experiments, the flow rate was 1 PV per 3 min. Red: grayscale binarization method, Blue: grayscale interpolation method. (**b**) Zoomed in ROS during the first 3 min (1 PV).

Figure 6 gives an overview of time-dependent ROS values obtained by flooding with HSW up to 1 day. Two successful experiments were performed, each with a ‘pristine’ calcite-coated micromodel (they were not re-used). In the first experiment, we study the temperature effect by flooding for 3 to 3.5 h before addressing the next temperature, going from 22 °C to 60 °C to 90 °C. This experiment ensures that we measure an effect of temperature increments only, since the distribution of the coated calcite remains the same. In the second experiment, we start at 90 °C and maintain that temperature for 24 h. A comparison with the first experiment can then be made. Examples of the CRO/brine distributions after HSW flooding are shown in Figure S11. Intermediate shades of brown are visible in each image.

We first consider the two different methods for calculating ROS. Clearly, the ‘binarized ROS’ and ‘interpolated ROS’ values are typically not the same in Figure 6, with differences lying in the range of 0–8% in the first experiment. There also appears no systematic bias which would cause one calculation method to yield higher/lower ROS than the other. Importantly, the qualitative trends (with time and temperature) appear to be the same for both methods. The grayscale histogram also displays the same trend. Predominantly, it is the histogram shape that changes during the waterflooding, i.e., going from 22 °C to 90 °C the CRO peak (grayscale ~100) gets lower while the main brine peak (grayscale ~180) gets higher. This clearly indicates that CRO has been replaced by brine (Figure S12). Small shifts in grayscale also occur; in particular the CRO becomes slightly less light-absorbing at higher temperatures. However, this effect was taken into account: in the binarization method by adjusting the threshold, and in the interpolation method by using calibration images taken at the temperature of interest.

Both ROS calculation methods indicate that small amounts of additional oil are released (the ROS decreases by a few percent) as the temperature increased from 22 °C to 60 °C and from 60 °C to 90 °C. We emphasize here that the transitions between the temperature regimes were mechanically ‘gentle’: the brine flow was maintained while the electric current to the heating block was increased gradually. The occasional small ‘humps’ in the ROS number correspond to sparse droplets of CRO that had initially remained stuck in the inlet of the pore space and were suddenly released; typically, they were transported through the pore space, after which the ROS value returned to its value before the release. Besides such rare events, practically no changes occurred on prolonged exposure to the brine flow at the same temperature (as shown in Figure 6a). This is not only indicated by the plateaus in ROS but also by signals calculated from grayscale difference images (see Figure S13). The ROS plateaus generally correspond to difference maps that are near-zero (i.e., within 10 grayscale values) for all pixels, suggesting that hardly any spatial exchange between brine and oil had taken place, and that the inflowing brine was principally displacing other brine. The predominance of the first brine-sweep of the pore space is also illustrated in Figure 6b, where the number of pore volumes needed to reach the ROS plateau is close to (1—ROS). In other words, all the injected brine is used to displace CRO until a continuous flow path for brine is formed, after which the newly injected brine follows that flow path, as is often observed [37].

Figure 6 shows that the effect of the waterflooding temperature on the residual oil saturation depends on temperature history. The solid lines show the experiment where the HSW flooding was initiated at 22 °C and the temperature was subsequently raised to 60 °C and 90 °C. The ROS decreases from 100% to ~57% after 3 h at 22 °C, decreases further to ~53% after 3 h at 60 °C and drops further to ~49% after 3 h at 90 °C. For comparison, the experiment where the waterflooding was initiated at 90 °C (dashed lines) reaches a ROS of 32/41% after 3 h. In other words, comparing the ROS plateaus at 22 °C and 90 °C within the 22-60-90 °C experiment, the ROS decrease is only ~8%. Meanwhile, comparing the initial ROS plateaus between the two experiments (at 22 °C for the solid lines and at 90 °C for the dashed lines), the difference is much larger at 16–25%. Hence, the qualitative trend is that ROS decreases with temperature, but the quantitative change is bigger if we compare the conditions of the first flooding.

The numerical ROS values measured with the two methods are also summarized in Table 1. In this table a comparison is also made with earlier experiments [37] conducted with pure glass micro-models having the same 2.5D pore network geometry. These experiments for which chip fabrication and characterization are less laborious indicated a typical ROS standard deviation of 3–5% [37]. Comparing the ROS values, as calculated using the binarization method, it comes out that the partially calcite-coated micromodels show a 6–10% higher ROS than the uncoated micromodels. This indicates that oil retention is enhanced by coating the glass with the combination of the photocured polymer and the calcite. The pristine photocured polymer appears to be slightly less hydrophilic than glass (see Table S4).

#### 3.2.2. Long-Time Shut-In Effect at Elevated Temperature

**Figure 7:**
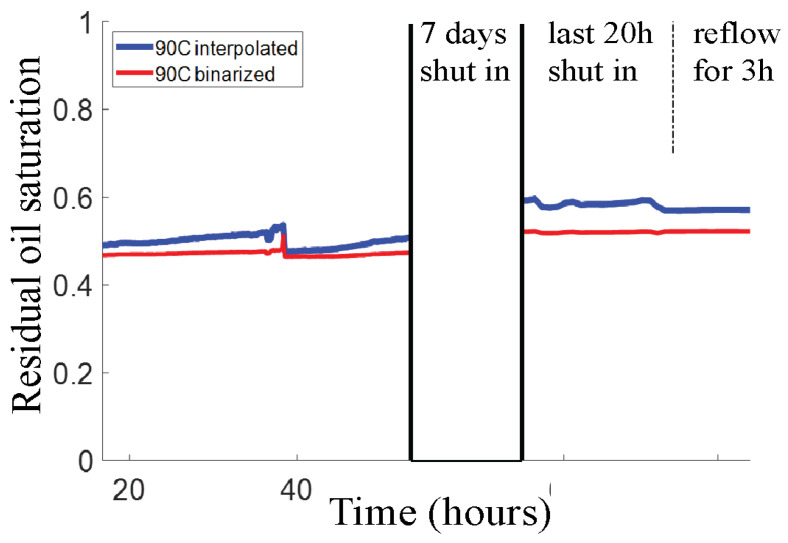
Long duration measurement of the residual oil saturation (calculated with two methods as before) at 90 °C. The labels on the time axis refer to the time lapse since the experiment was started at 22 °C (see Figure 6a).

**Figure 8:**
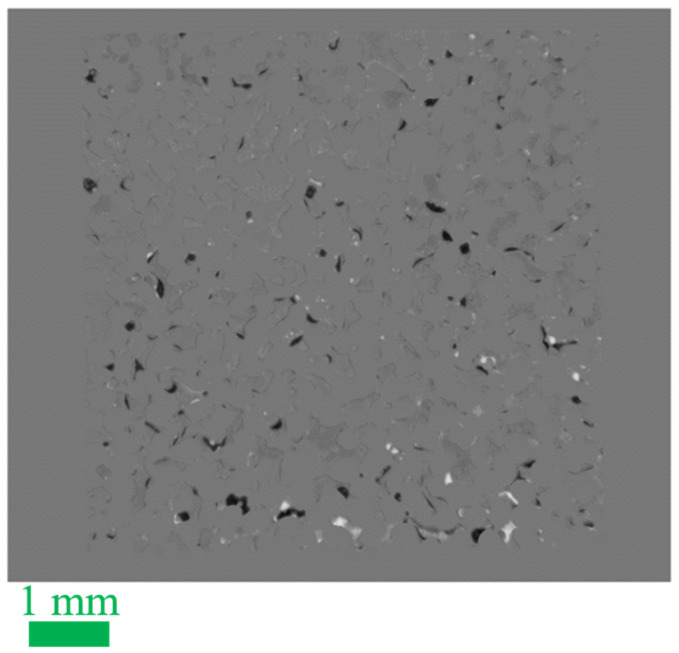
Grayscale difference image ∆I(x,y) = I2(x,y) − I1(x,y), mostly revealing changes in the distributions of brine and oil in deep pores before (I1) and after (I2) the 8 days of exposure to HSW at 90 °C in absence of flow. In absence of changes (e.g., the glass parts), the pixels show a gray value of 120. Darker areas (gray value 0–119) indicate the replacement of brine by CRO, while it is vice versa for the brighter pixels (gray value 121–255). Further details on the calculation and presentation of the grayscale difference image can be found in Figure S10.

To look for the possibility of slower processes, we extended the final 90 °C phase of our 22-60-90 °C experiments to much longer times. After flooding with HSW for ~40 h at 90 °C, the flow was stopped for 8 days, after which it was resumed for 3 h at the standard rate of 0.2 µL/min. The ROS evolutions are shown in Figure 7. Remarkably, the changes in calculated ROS values are rather modest. After the long shut-in, the calculated ROS appears to be a few percent higher. However, visual analysis of the image-time series did not reveal any flow of CRO into the pore space after the flow was stopped, as expected. A grayscale difference map that compares the occupancies of the pore space before and after the shut-in does show a modest redistribution of brine and CRO: see Figure 8. The apparent small ROS increase over the ‘shut-in’ might partly be attributed to the release of CRO from the shallow pores (see Figure S9). Finally, restarting the flow does not produce any change in ROS.

**Figure 9:**
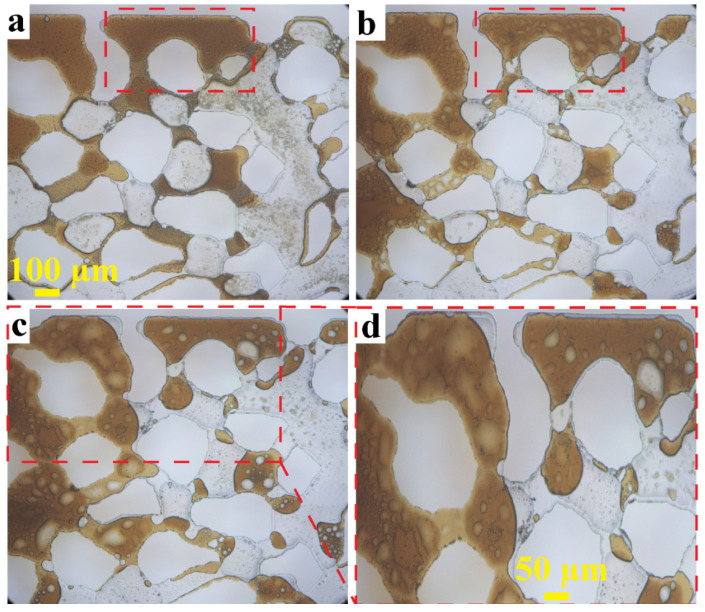
Aqueous drop formation and swelling during exposure of CRO to high-salinity water: (**a**) after 3.5 h at 22 °C, (**b**) after an additional 48 h at 90 °C (**c**) after a shut-in period of 8 days, (**d**) Zoom-in of a part of panel (**c**). Red boxes illustrate regions where the swelling of brine drops gradually occurs over time.

We further examine the effect of long-time exposure to HSW at 90 °C by visualizing some areas of the pore space at higher (i.e., 10×) magnification. Figure 9 shows a growing prominence of brine slugs over time. Small aqueous droplets are present at all stages, but the extent of the swelling of the brine droplets is spectacular, with some slugs spanning more than 50 µm. Since the slugs show less brightness compared to pure brine, they must be coated by a CRO film, at least on one side. How these large slugs were formed, could not be inferred from our images (which were taken only intermittently at 10× magnification); we will consider possible mechanisms in the discussion. Occurrence of most of the oil-immersed water in the form of large slugs rather than dispersed microdrops might perhaps explain why the overall ROS of the pore space does not get lower and the sweep efficiency is not increased.

### 3.3. Effect of Calcite Particles

Accurate assessment of the influence of calcite on the residual oil saturation is not trivial in the case of a partial coating with calcite particles. It starts with an estimate of the fraction of the inner chip surface that is coated with calcite. In our case, where calcite nanoparticles were used as seeds, we assume that the majority of them grew large enough to become visible with optical microscopy. Presence of calcite particles is indeed observed at 10× magnification images (see Figure S6). However, from such images it also becomes clear that the spatial distribution of the calcite is not entirely homogeneous: the pore edges appear richer in particles as compared to the centers of the pores. It is more difficult to quantify the amount of calcite in the edges, because the non-horizontal pore walls give a more complex projection image. The pore centers do not suffer as much from this effect, and offer a more flat substrate. Therefore, we analyze the ROS evolutions separately for the pore centers and the pore edges.

**Figure 10:**
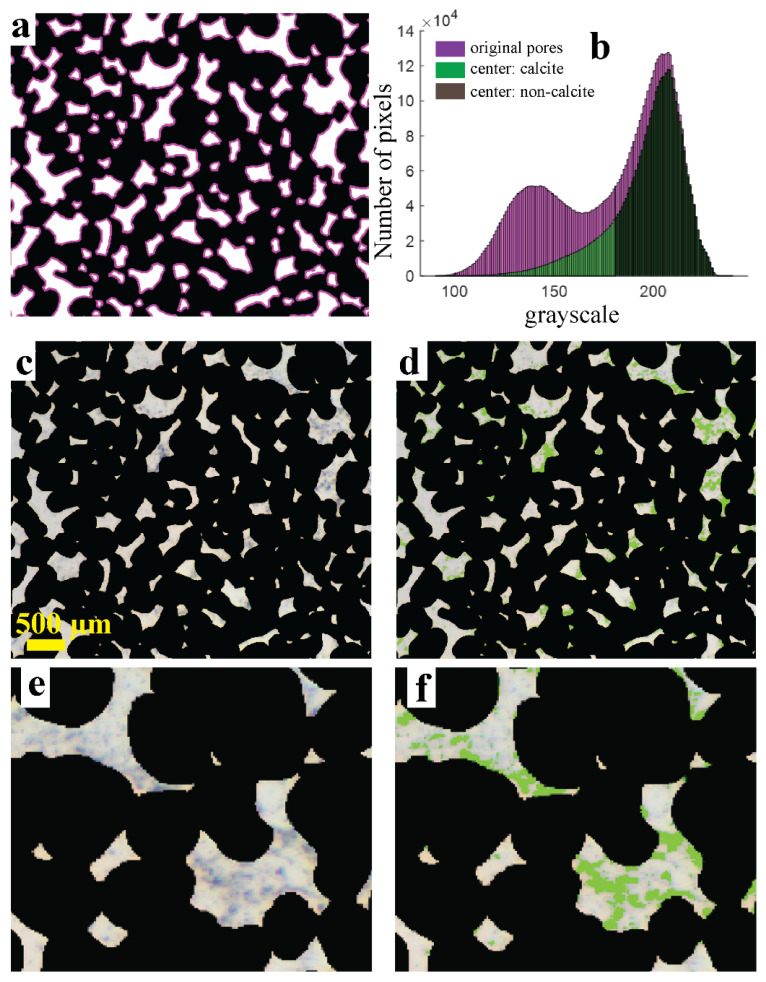
(**a**) False color map representing the erosion of deep pores, to obtain a separation between pore centers and pore edges. The magenta parts become black in the new overlay mask for the pore centers. (**b**) Application to the brine-filled chip shows that most of the secondary peak is removed. (**c**,**d**) Identification of calcite particles in the pore centers, by green-marking those pixels in the left image for which 0 < grayscale value < 180. (**e**,**f**) Zoom-in of a part of panels (**c**,**d**).

Figure 10a shows this division of the deep pores in edges (magenta) and centers (white) for an edge width of 24 µm; choosing the edges to be slightly narrower or wider does not affect the qualitative outcome of the ROS comparison between the different pore regions. The histograms (obtained from the FW-filled chip) in Figure 10b (original pores vs. centers) confirm that a significant fraction of the dark pixels is located at the edges. For the pore centers, a sub-classification into calcite and non-calcite pixels was made by choosing a grayscale threshold while visually comparing images, such as Figure 10c,d. This produces a calculated calcite coverage of ~20%, with an estimated error of 5%. The precise choice of grayscale threshold (we used 180) does not affect any of our qualitative conclusions.

**Figure 11:**
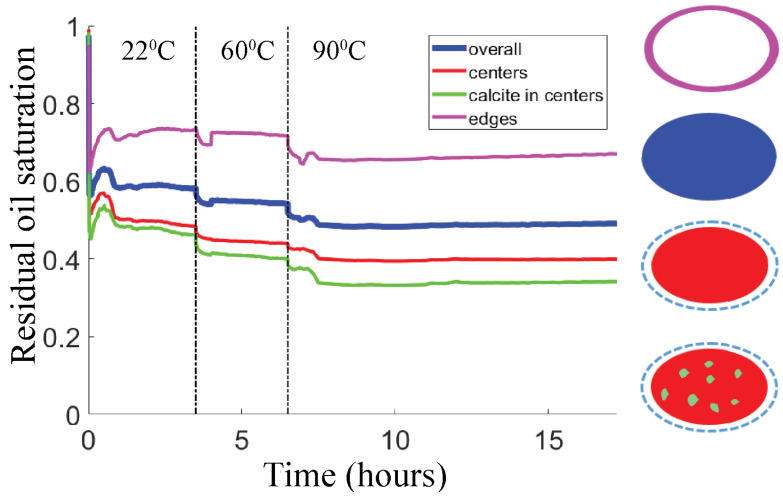
(**Left**) Splitting of the overall ROS (blue, also shown in Figure 6a) into contributions from the centers (red) and the edges (magenta) of the pores. For the pore centers, the ROS as measured on calcite-coated areas (green) is also shown. Pore centers, pore edges and calcite areas were defined as shown in Figure 10. (**Right**) Description of pore division.

In Figure 11, the ROS trends in the 22-60-90 °C waterflooding experiment are compared for different subsets of the pore space. The ROS values are calculated using the interpolation method, which better accommodates for the effect of calcite on the local grayscale value. Clearly, the ROS values measured in the pore edges are higher than the ROS values in the pore centers. The difference in ROS between the overall pore centers and only the calcite-coated parts of the pore centers is less clear: the green curve falls everywhere below the red curve, but the differences are relatively small.

**Figure 12:**
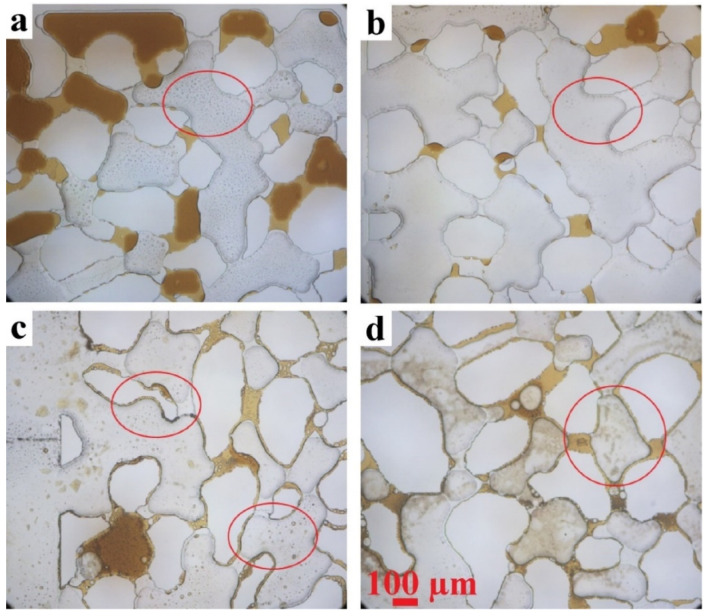
Comparison of the oil-retaining tendencies of the pore edges in (**a**,**b**) glass (ref. [37].) and (**c**,**d**) calcite-coated microchannels, at 90 °C and using HSW as the brine. Red circles show the differences in edges with and without retention of CRO.

Similar trends are found for the 90 °C waterflooding experiment (see Figure S14). High-magnification (10×) still images from this experiment are shown in Figure 12c,d. Clearly, the CRO is preferentially retained at the pore edges, in agreement with the higher ROS values measured from the low-magnification (2×) images. Even 20 h of exposure to flowing HSW does not lead to the removal of this local CRO. The high magnification images thus confirm the trend in Figure 11 that the calcite particles in the pore edges are more effective in retaining CRO than the particles residing on the flat walls.

In our earlier work using the same aging and flooding conditions and microchannel geometry but without the calcite functionalization, we found that CRO mostly “dewets” from the glass pore walls at 90 °C, ref. [37] and CRO is less retained by the pore edges; see Figure 12a,b. The calcite functionalization thus has a strong effect on CRO retention at the pore edges. To make the comparison more quantitative, we calculated the fraction of edges in which the CRO is retained. In the calcite-coated microchannel, ~80% of the edges retains CRO, while it is only ~20% in the glass microchannel.

## 4. Discussion

One key parameter that was varied in our waterflooding experiments is the duration of the interaction between brine, CRO and the pore surfaces. In previous works (mostly using glass micromodels), different time scales of the brine injection were reported. In several studies, short durations up to 1 h were found to be sufficient for recovering a significant fraction of the original oil in place, and the duration was not extended beyond one to a few hours [5,6,45]. However, processes such as wettability alteration, solid–liquid interface interaction and brine/oil swelling, were also found to evolve over several days in several studies [34,35,38,46]. In our work, time scales from less than 1 min to more than 1 week are addressed. Exposing our micromodels to HSW at 90 °C, we found that after the ‘immediate effect’ of flooding, only a very small amount of additional CRO gets released, in spite of flooding more than 900 pore volumes during 2 days, and interrupting the flow for 8 days before resuming the flow. Incidental ~1% reductions in ROS are likely due to oil slugs that were at the brink of getting released and needed just a ‘critical fluctuation’ in pressure. Hence, our findings do not give any indication for a slow wettability alteration (as could perhaps be expected for an ongoing desorption process).

Stepwise increases in the waterflooding temperature, while sustaining the HSW flow, lead to short-time ROS reductions of 3–5%, for both the 22–60 and 60–90 °C steps. These changes are not artifacts. Although the two methods for calculating ROS can generate differences exceeding 5% for the same brine/CRO distribution, the relative ROS changes calculated within the same method are more accurate. The small temperature dependence of the grayscale values was taken into account in all calculations. Additionally, grayscale histograms (See e.g., Figure S12) confirm that some additional oil was released. The temperature effect might be due to changes in interfacial tension(s). A temperature-induced wettability alteration of calcite-coated surfaces was reported in a previous study upon injection with NaCl brine at temperatures up to 70 °C [47]. Gupta et al. observed a similar alteration on limestone rock. They used contact angle measurements to show that brines with sulfate and calcium ions render the limestone surface more water-wet at 70 °C and 92 °C compared to RT [48]. The temperature effects obtained in this study agree with the results of several core scale studies that reported that HSW can improve the water-wetness of carbonates at high temperatures based on spontaneous imbibition experiments [49,50]. Such findings also indicate the representativeness and scalability of presented results from calcite-coated micromodel to the core scale.

Significant observations were also made from high magnification (i.e., 10×) images, taken at various stages of the HSW flooding. Dark structures near the brine–CRO interface (as already seen in the 2× images, Figure 5a) and dark structures immersed in bulk oil (Figure 9), are due to small (1–5 µm) aqueous droplets immersed in the CRO. These so-called aqueous micro dispersions have more often been found in EOR studies [51,52,53], and are formed at short timescales (within hours) at all explored temperatures. At 90 °C and much longer times (days to a week), we also observed the formation and growth of large (10–100 µm) ‘blisters’ (Figure 9b–d).

Water-in-oil micro-dispersions have been invoked to explain the effects of low salinity in IOR [45,54] and alkaline flooding [55]. Bartels et al. [56] observed the presence of water/oil structures in X-ray micro CT experiments during high-salinity waterflooding at RT. However, it could not be verified whether these structures were (micro) emulsions, due to the spatial resolution of the technique. Fredriksen et al. [57] visualized water-in-oil emulsions due to osmotic water movement and coalescence into larger droplets during Low Salinity Waterflooding. Recently, Selem et al. [58] observed a clear development of water droplets within the oil phase from high salinity to low salinity flooding at 70 °C in a carbonate rock sample by micro-CT.

**Figure 13:**
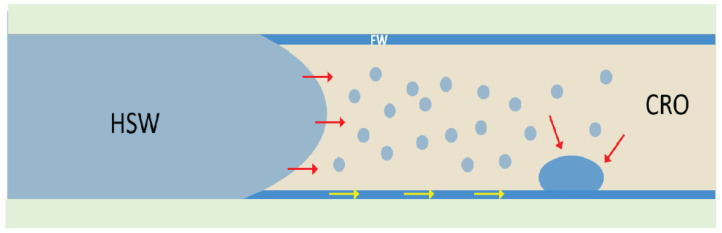
Schematic illustration of possible ways in which the aqueous blisters shown in Figure 9 can grow. A thin film of formation water with very high salinity that was left behind during the aging, attracts water from other sources where the water chemical potential is higher. Water molecules can be transported through the CRO phase (red arrows), and/or directly through the thin brine film (yellow arrows).

In our case, it remains a question whether the blisters originate from the micro dispersion droplets or not. Coalescence of micro dispersions has been observed in brine/CRO systems [53] but generally requires either: (i) that they were never thermodynamically stable, like most emulsions that are prepared via mechanical agitation; or (ii) that the stabilizing surface active species are removed from the interface. In our case, the aqueous microdroplets were formed spontaneously, and presumably there was no subsequent change in the chemical potential of the water since contact with HSW was maintained. Given the chemical equilibrium with respect to the water, i.e., µ_H_2_O_ being the same in the HSW, the CRO and the microdroplets, there would not be a driving force for the microdroplets to grow to the large slugs that were observed after several days. However, if a thin film of formation water would have remained throughout the aging and waterflooding stages, a driving force for the swelling of aqueous slugs would exist. Due to its higher salinity, FW has a lower µ_H_2_O_ than HSW and would therefore attract water from all sources that have the same µ_H_2_O_ as HSW: the bulk HSW, the microdroplets or the molecularly dissolved water in the CRO. This scenario is sketched in Figure 13. The swelling would stop at the point where the FW was sufficiently diluted with lower salinity water, to match the equilibrium µ_H_2_O_.

Hence, a similar ‘osmotic’ swelling in the reported previous studies [57,58,59,60] would occur, but now involving HSW as the ‘lower salinity’ brine. In our sketched picture, the aqueous blisters are thus formed at a pore wall. The effect of the blisters on the release of CRO appears to be insignificant. Simultaneously with the growth of the brine slugs, the CRO also appeared to accumulate in some of the pores. These CRO slugs were not pumped out after the flow was resumed.

Considering that our partially calcite-coated micromodel has the same pore network geometry as used in our earlier study where the chip was purely made from glass, it is interesting to compare the outcomes. Some trends are similar, such as the dominance of the first sweep: the vast majority of the CRO gets removed within the first pore volume. Also, a higher first-flooding temperature results in more produced oil in both cases. The ROS values appear to be higher for the calcite-coated chip. However, a comparison between the two micromodels involves more than simply the physico-chemical properties of ideal glass and calcite surfaces. The presence of a partial coating by calcite particles can introduce heterogeneities in (i) the wettability compared to the non-coated substrate and (ii) the spatial distribution of the particles. The latter was verified to be the case: after removing the weakly bound calcite particles (see Section 2.2), the pore edges were found to be coated more densely than the pore centers. Separate analysis of the ROS trends in the edges and the pores revealed that the high overall ROS (compared to the glass chip) is largely due to the pore edges, where not only the chemistry, but also the local (roughness) geometry, was modified by the calcite particles. It is known that roughness enhances the pinning of contact lines [61].

The lack of a clear effect of the calcite particles in the pore centers might result from different origins. The wettability contrast between the aged calcite and the aged adhesion layer (photocured polymer) might be small; for example, if both are ‘cloaked’ with a layer of adsorbed CRO molecules during aging. Even if the wettability contrast is significant, the low coverage and small roughness (which are correlated to each other) of the calcite particles might be insufficient to cause a strong contrast in local ROS. Surface roughness is omnipresent in real rock structures due to the diagenesis process, but only few microfluidic studies investigated its effect on two-phase fluid flow in oil recovery [62,63,64]. In one of them, it was reported for glass micromodels [62] that a pore-scale surface roughness (defined as ratio of hillock height to pore depth) less than 12.5% results in a minor impact on the sweep efficiency when injecting air into a crude-oil-saturated micromodel. In our micromodel, the calcite coverage density was crudely measured to be ~20% based on a projection image that captured both the floor and the ceiling of the pore space. The calcite particles within the clusters are ~1–2 µm, comparable to the surface roughness regime addressed in previous study [62].

The ability of the calcite-rich edges of the pores to more strongly retain the CRO is indicated by the ROS trends and confirmed by the high-magnification images that showed layers of CRO adhering to the sidewalls. The strong contrast with the all-glass chip, where the pore edges showed a clear dewetting at 90 °C must be due to a combination of the different chemistry and geometry of the pore edges. Further experiments are required to detangle the individual contributions of surface chemistry and topography in driving oil displacement within pore networks.

Our current findings also indicate possibilities for further developing both the micromodels and analysis methods for IOR from heterogeneous reservoirs. The calcite coverage density of the pore space could be increased by further optimizing the seeding and growth of the nanoparticles, or perhaps by using atomic layer deposition to obtain a more homogeneous calcite layer, although this has not yet been realized in microfluidic chips (as far as we know). Incidental release of small CRO slugs from the inlet channels could be mitigated by using separate entrances for oil and brines.

Our data analysis showed that the precise calculation of ROS values can become challenging for two reasons: (i) optical heterogeneities due to calcite particles; (ii) coexistence of brine and oil in the same pixel. In such cases, use of a ‘single grayscale’ binarization to classify pixels as either brine or CRO filled, is incorrect. Our interpolation method can improve such situations by calculating a ROS per pixel, while also taking into account intrinsic differences between pixels (calcite-coated or not). Potentially, our pixel-based ROS ‘interpolation’ method can also be used to detect local disappearances of calcite particles due to dissolution, which is of interest in waterflooding experiments at low salinity. The occurrence of light scattering (by formed microdispersions) cannot be accommodated by either the binarization or the interpolation method. For these reasons, we recommend comparing the outcomes of different methods of ROS calculation, and to use additional diagnostic image analysis (such as difference maps) to obtain a better perspective on the consistency of the outcomes.

## 5. Conclusions and Outlook

By integrating and optimizing a hybrid of existing methods, we achieved a 2.5D glass microchannel, which is partially coated with calcite and mimics the dual porosity of carbonate rocks. The calcite coverage density can be controlled via the amount of nanoparticle seeds for crystal growth and the number of exposures to a supersaturated solution. Spectroscopic analysis confirmed that pure calcite is grown on the glass surface, and optical microscopy under flow indicated their mechanically stable attachment. To determine the local amounts of brine and oil in the pore space, a novel image analysis method was developed, which takes into account the darker appearance of the calcite particles (compared to uncoated areas) in two ways: (i) it localizes calcite-rich areas and (ii) it calculates a fractional ROS per pixel.

The qualitative effect of the temperature of HSW flooding on the residual oil saturation looks similar for our partially calcite-coated microchannel and a bare glass channel with the same pore network. Another similarity is that, after the water breakthrough, only minor subsequent changes in overall ROS occur at the timescale of hours. Exposing our calcite-coated chip to HSW at 90 °C for much longer times (several days to a week), a spectacular formation of large aqueous blisters is observed. However, this process leads to a redistribution of water and oil within the pore space, rather than the production of additional oil.

The overall effect of the partial calcite coating on CRO retention appears to be a modest increase in ROS. On flat surfaces, areas that are partially coated with calcite (about 20% surface coverage) do not appear to retain oil more effectively than their surrounding “bare” substrate. At the more calcite-rich pore edges, a significantly higher ROS is found, but the possible effect of calcite chemistry cannot be disentangled from the effect of wall roughness introduced by the particles.

Based on the outcomes of this work, we expect that lowering the salinity of the flooding brine will have an effect on several processes that together determine the amount of CRO that ultimately gets released. The formation of microdispersions and blisters are both driven by the water chemical potential, which is higher for low salinity brine. In addition to this, the solubility of calcite in low salinity brine can cause strong changes in wettability. Additional diagnostic methods for detecting in situ calcite dissolution may need to be developed.

## Figures and Tables

**Table 1:** Residual oil saturation (ROS) values measured after flooding the calcite-coated chip with high-salinity water at different temperatures and for short and long times. Alongside ROS values obtained from all-glass chips with the identical pore network are shown [37]. ROS values for the all-glass chip are only available for the binarization method; the interpolation method produces the same trends. NA means ‘not available’.

	Binarization Method		Interpolation Method	
Experiment	Short Time	Long Time	Short Time	Long Time
	(0.5–1 h)	(24–48 h)	(0.5–1 h)	(24–48 h)
Calcite chip at 22 °C	~56%	NA	~58%	NA
continued at 60 °C	~51%	NA	~55%	NA
continued at 90 °C	~48%	~46%	~50%	~49%
Calcite chip at 90 °C	~41%	~40%	~32%	~32%
Glass chip at 22 °C *	46 ± 3%	NA	NA	NA
Glass chip at 90 °C *	35 ± 5%	NA	NA	NA

(*) All-glass chip with identical pore network.

## Data Availability

The data supporting the findings of this study are available from the corresponding author upon reasonable request.

## Supplementary Materials

The Supporting Information can be downloaded at: https://www.mdpi.com/article/10.3390/mi13081316/s1. Table S1: Composition of Formation Water and High Salinity water. Table S2: Physicochemical characteristics of crude oil. Table S3: IFT of High Salinity water with CRO at different temperatures. Table S4: Contact angle of HSW in air on different surfaces on glass substrate. Figure S1: Etch masks of the 2.5D microchannel. Figure S2: Reconstructed image of connectivity of deep and shallow pores. Figure S3: Calcite nanoparticle inside the glass microchannel. Figure S4: Growth of CaCO3 particles. Figure S5: Experimental setup. Figure S6: A section of calcite-coated microchannel after FW aging step. Figure S7: Evolution of average grayscale intensity of pixels covered by glass. Figure S8: Grayscale histogram for the shallow pores. Figure S9: Residual oil saturation for shallow pores. Figure S10: Detail explanation of grayscale difference image. Figure S11: Brine/CRO distribution images. Figure S12: Grayscale intensity histograms at different temperature stages. Figure S13: Grayscale difference maps. Figure S14: ROS at different contribution for experiment directly at 90 °C.
