# Supplementary material for: Oil Displacement in Calcite-Coated Microfluidic Chips via Waterflooding at Elevated Temperatures and Long Times
Source: Micromachines (Basel). 2022 Aug 14;13(8):1316. doi: 10.3390/mi13081316 (PMC9415086; doi:10.3390/mi13081316)
Supplement: Supplementary file 1 [file micromachines-13-01316-s001.zip › micromachines-1839758-supplementary.pdf]

micromachines

Supporting Information

Oil Displacement in Calcite-Coated Microfluidic Chips via Waterflooding at Elevated Temperatures and Long Times

Duy Le-Anh [1,*], Ashit Rao [1], Amy Z. Stetten [1], Subhash C. Ayirala [2], Mohammed B. Alotaibi [2], Michel H. G. Duits [1,*], Han Gardeniers [3], Ali A. AlYousef [2] and Frieder Mugele [1]

1 Physics of Complex Fluids, MESA+ Institute, Faculty of Science and Technology, University of Twente, P.O. Box 217, 7500 AE Enschede, The Netherlands
2 The Exploration and Petroleum Engineering Center-Advanced Research Center (EXPEC ARC), Saudi Aramco, Dhahran 34465, Saudi Arabia
3 Mesoscale Chemical Systems Groups, MESA+ Institute, Faculty of Science and Technology, University of Twente, P.O. Box 217, 7500 AE Enschede, The Netherlands
* Correspondence: a.d.le@utwente.nl (D.L.-A.); m.h.g.duits@utwente.nl (M.H.G.D.)

Table S1. Composition and geochemical analysis of artificial formation water (FW) and High salinity water (HSW).

	**Formation Water**	**High salinity water (HSW)**
Na^+ (ppm)	59491	18345
Ca^{2+} (ppm)	19040	650
Mg^{2+} (ppm)	2439	2117
Cl^- (ppm)	132060	35534
SO_4^{2-} (ppm)	350	429
HCO_3^- (ppm)	354	696
Total dissolved solids (ppm)	213734	57872
Ionic strength (M)	4.3	1.1
pH	6.8	7.2

Table S2. Physicochemical characteristics of crude oil.

**Viscosity (at 21.5°C)**	24	mPa ·s
**Acid number**	0.2 ± 0.04	mg KOH / g
**Base number**	1.1 ± 0.1	mg KOH / g
**Asphaltenes**	2.6 ± 0.3	mass %
**C10+**	91 ± 3.3	mass %
**n-heptane**	0.9 ± 0.4	mass %
**n-octane**	1.0 ± 0.2	mass %
**n-hexane**	0.6 ± 0.5	mass %

Table S3. IFT (mN/m) of HSW with CRO at different temperatures. Viscosity (mPa.s) of CRO and water at different temperatures.

Temperature	22 °C	60 °C	85 °C
IFT: HSW and CRO	3.2 ± 0.7	6.7 ± 0.2	7.6 ± 0.4
viscosity: CRO	24	7.4	3.4
viscosity: water	~1.0	~0.47	~0.34

IFTs between CRO and brine are generally time-dependent; they decrease over the first few minutes and then saturate. We present the saturated values measured after 20–25 min.

Table S4. Contact angle of HSW in air on different surfaces on glass substrate.

	Surfaces on glass substrate	Sessile drop CA	Advancing CA	Receding CA
HSW in air	Glass slide	40° ± 2°	42° ± 0.5°	19° ± 0.5°
	Spin coat "precursor liquid in IPA" on glass slide	46° ± 1°	47° ± 1°	27° ± 2°
	Spin coat "precursor liquid in IPA" on glass slide + UV curing	43° ± 1°	43° ± 0.5°	27° ± 2°

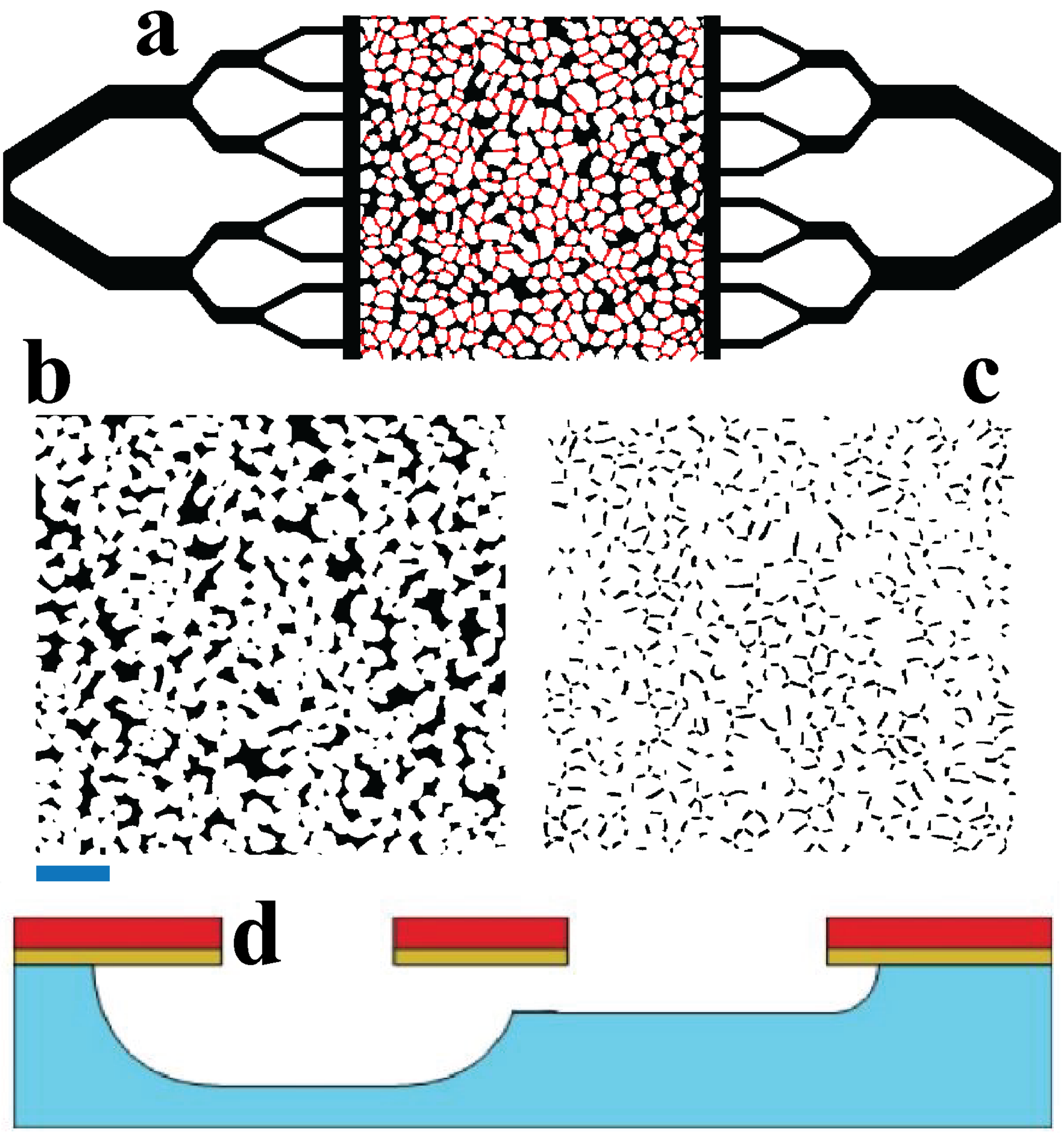

Figure S1. (a) Etch masks of the 2.5D micromodel, showing both the pore network and the distribution fractures. The black regions correspond to deep pores and channels and the red regions are the shallow pores. Middle row: (b) deep and (c) shallow pores separately. Reproduced with permission from ref [42]. Copyright [2019] Publisher Elsevier. Panel (d) shows a schematic side view of one deep and one shallow pore after the isotropic etching. Pore depths are 27 and 12 μm.

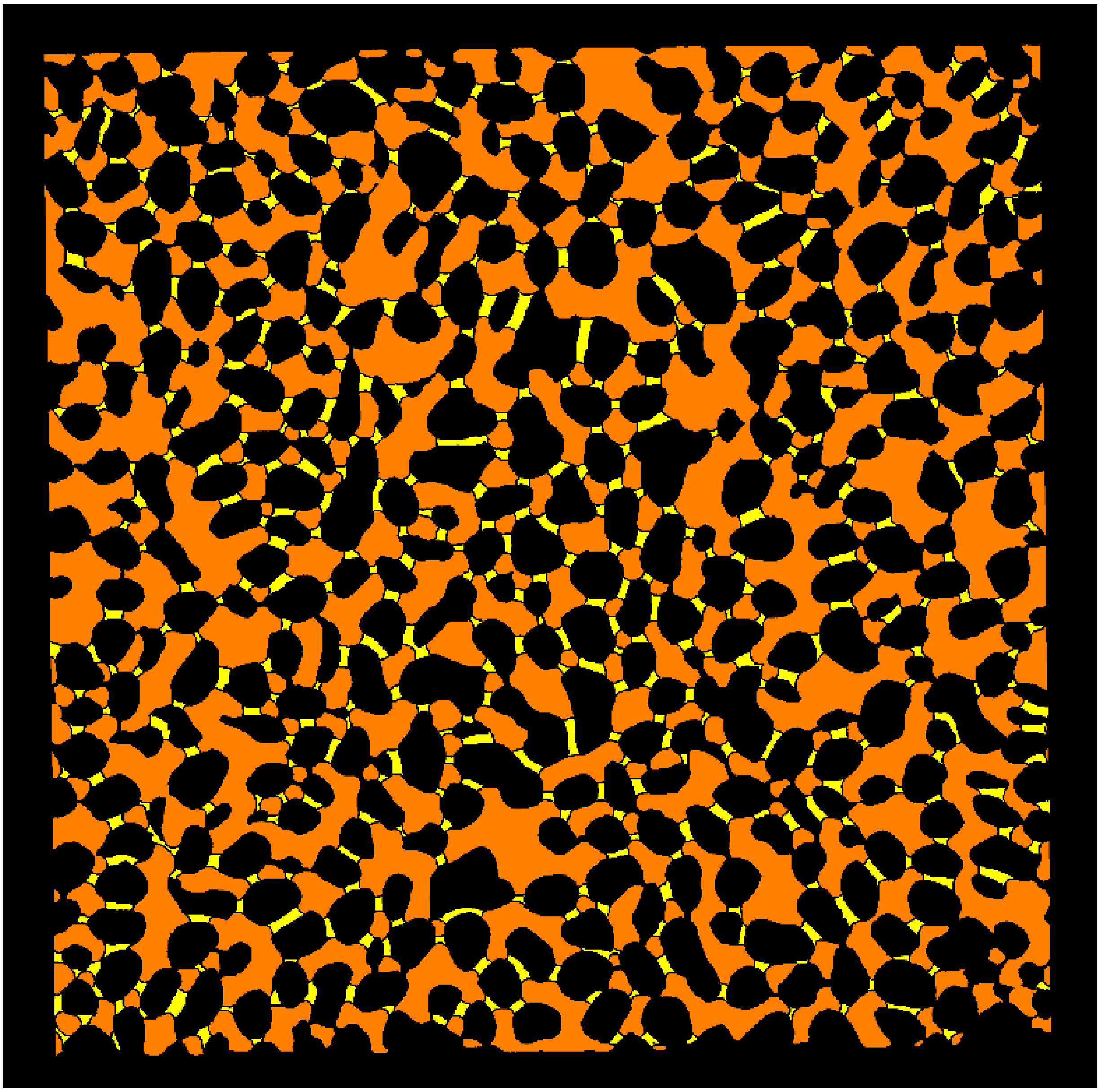

Figure S2. Reconstructed image highlighting the connectivity of the 'deep' pores (orange) and 'shallow' pores (yellow). This image is based on the snapshot of the CRO-filled chip shown in Figure 3b in the main text.

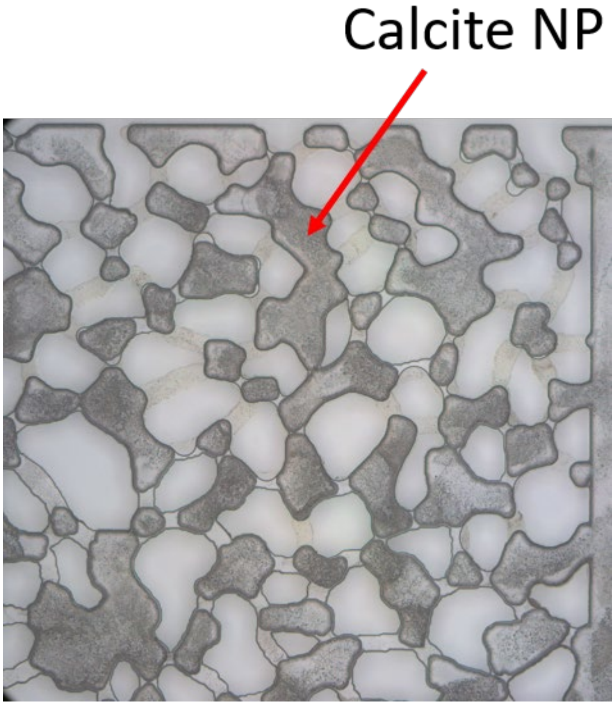

Figure S3. Dense calcite nanoparticle seed layer in a region inside the glass microchannel. Image taken after 3 cycles of calcite NP deposition.

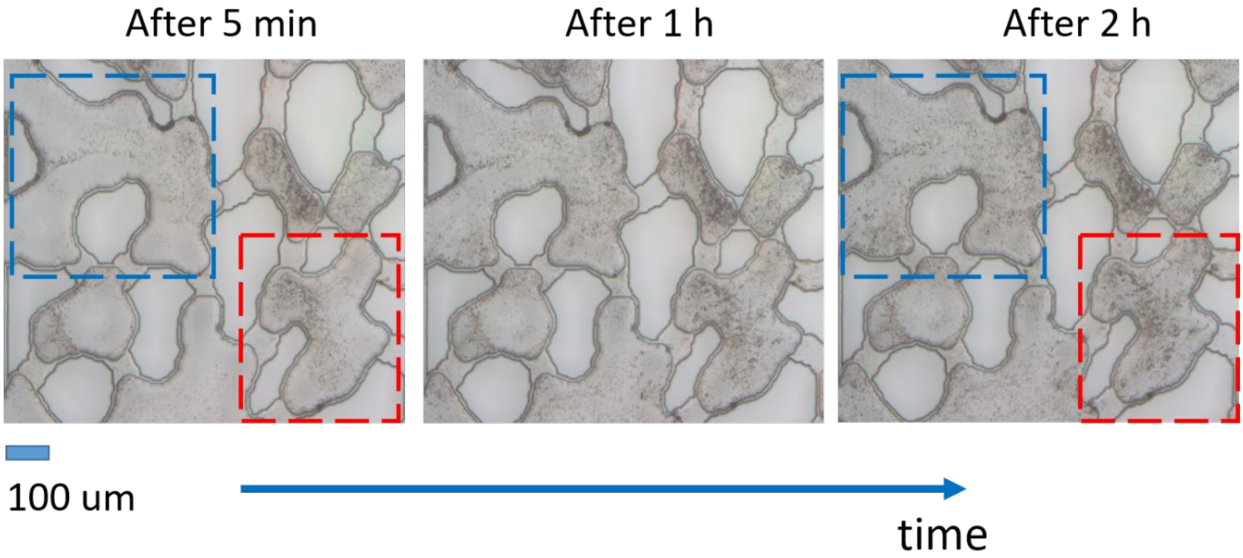

Figure S4. A section of microchannel: growth of the $CaCO_3$ with supplying more Ca^{2+}, CO_3^{2-} ions supersaturated solutions. $CaCO_3$ grows over time (Left) after 5 min, (middle) after 1 h, and (right) after 2 h. Supersaturated solutions was replaced every 30 min.

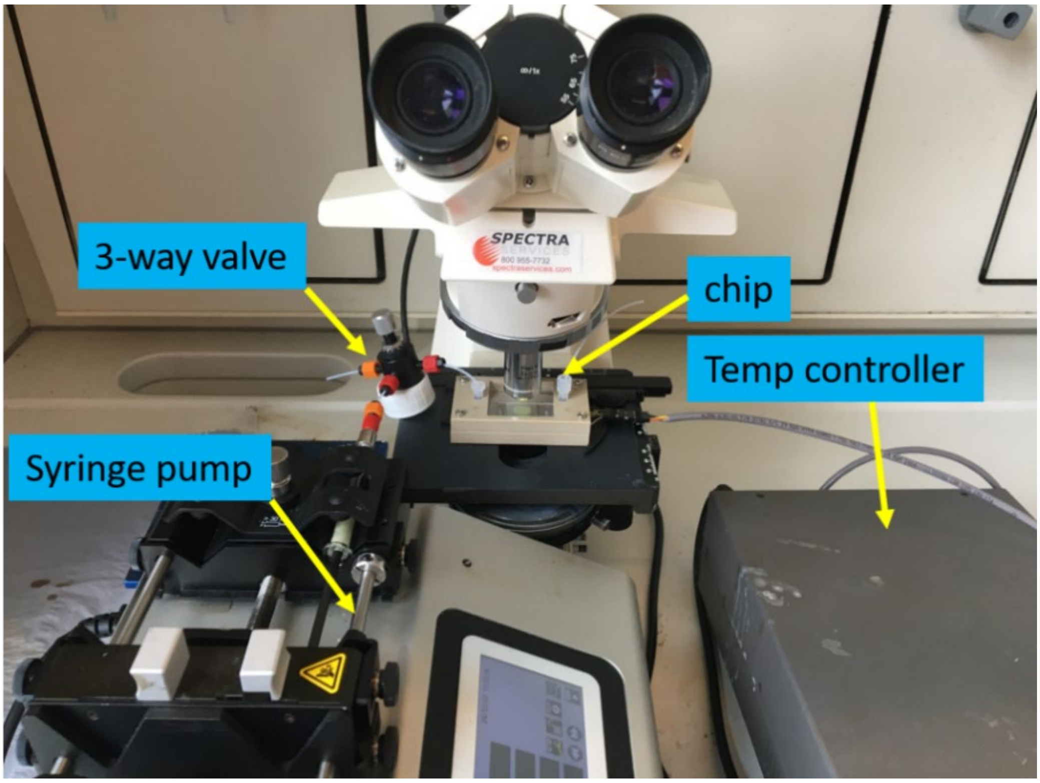

Figure S5. Experimental setup used in the aging and waterflooding experiments. Reproduced from ref [37] under CC-BY license.

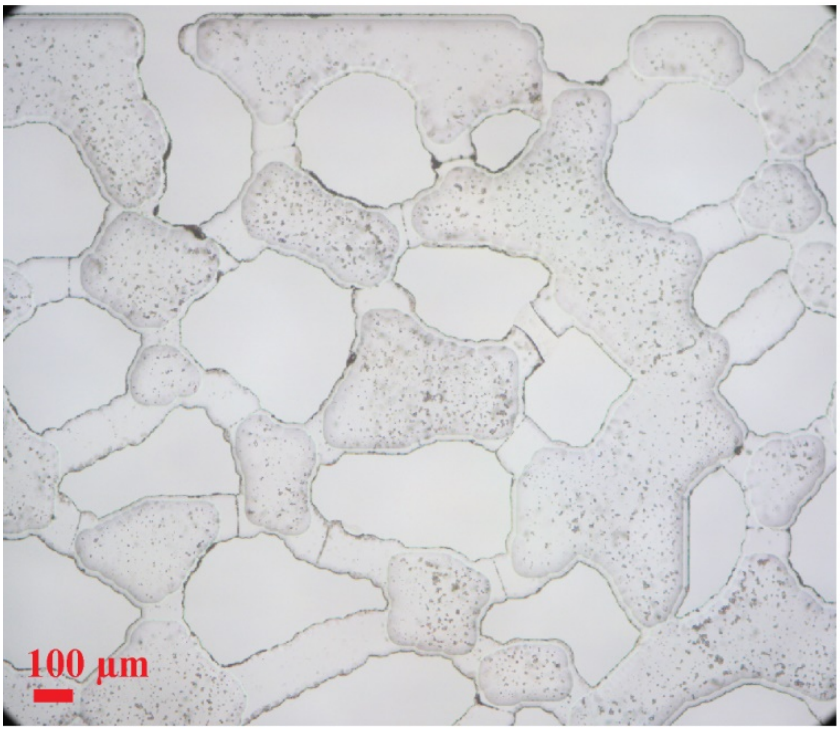

Figure S6. A 10X zoom in section of the calcite-coated microchannel used in the first experiment. Image taken at FW aging step, after strong FW flushing to remove weakly bound particles.

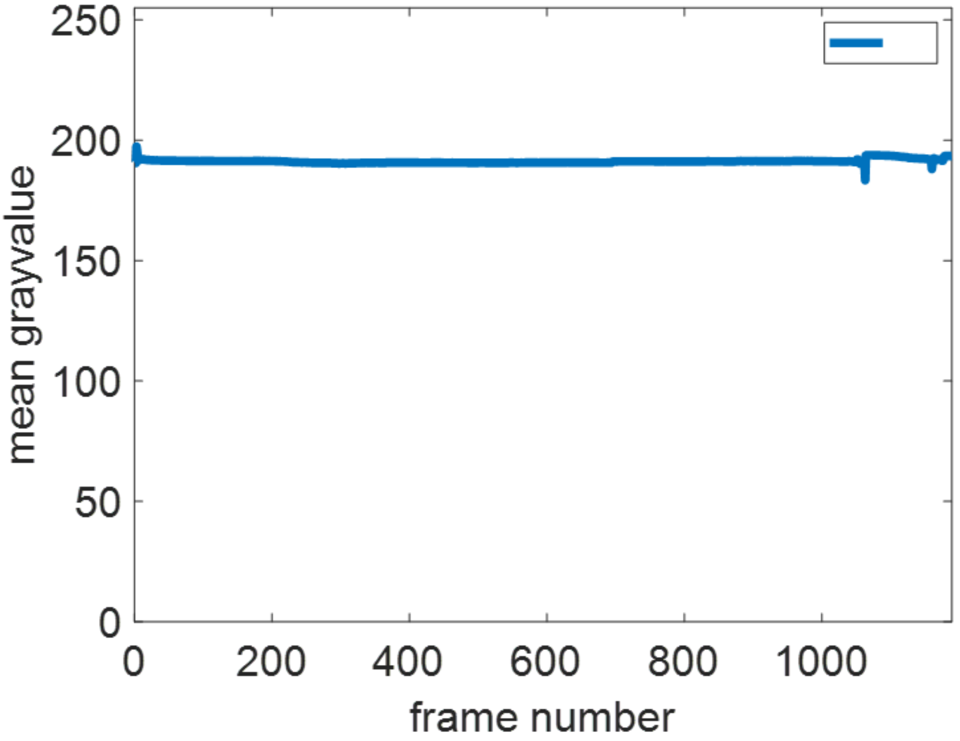

Figure S7. Evolution of the average grayscale intensity of the pixels that are covered by glass, for the 22-60-90C experiment shown in Fig. 6. The time interval between subsequent frames is not constant.

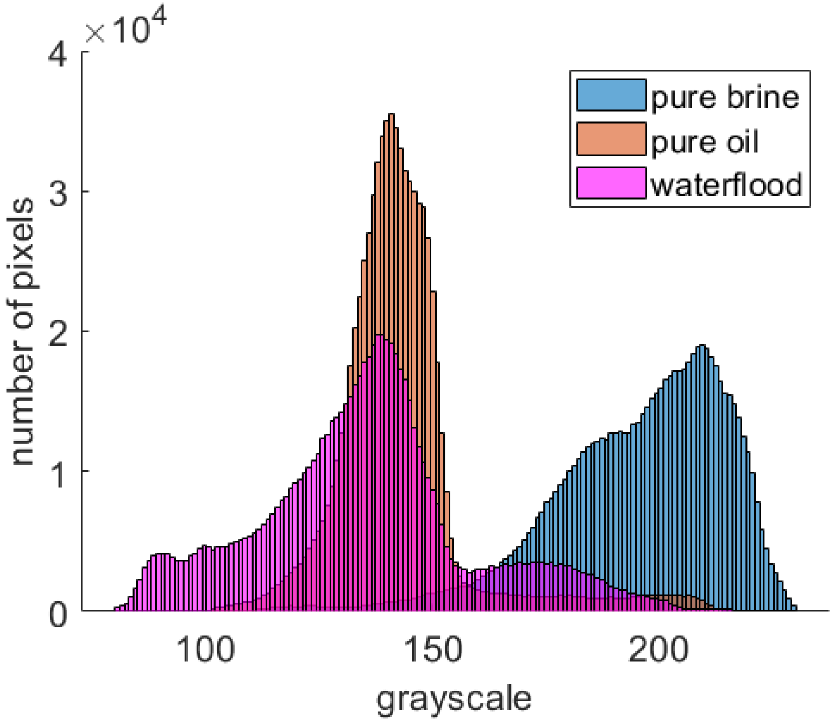

Figure S8. Grayscale histograms for the same experiment as presented in Fig. 5 for the shallow pores. The first 30 min of flooding with HSW at 22°C (magenta) is compared to the calibration data for brine (blue) and oil (brown).

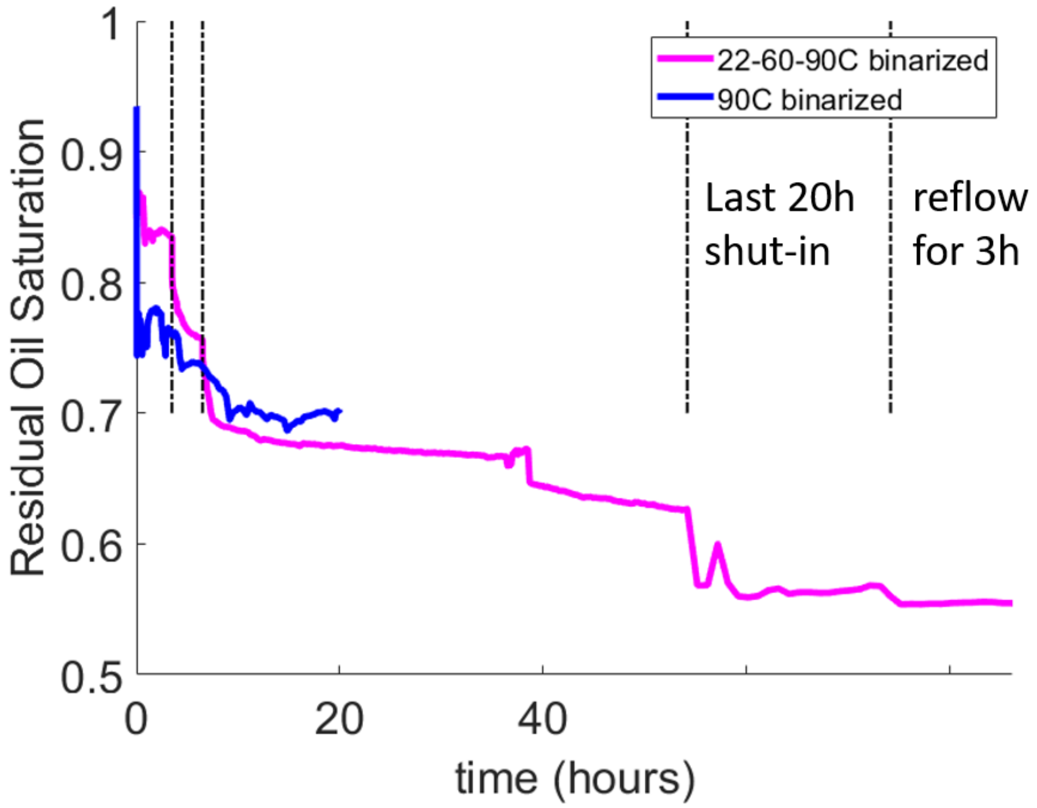

Figure S9. Data from the same experiments as shown in Fig. 6 in main text, but now for the shallow pores. Because ~10% of the shallow pores did not get filled with CRO during aging, only the binarization method (grayscale threshold: 160) was used to calculate ROS, and the initial ROS is ~0.9. Vertical Dashed lines indicate transitions in the 22-60-90 °C experiment; from left to right: 22 to 60°C, 60 to 90°C, ending of shut-in at 90 °C, resumption of flow.

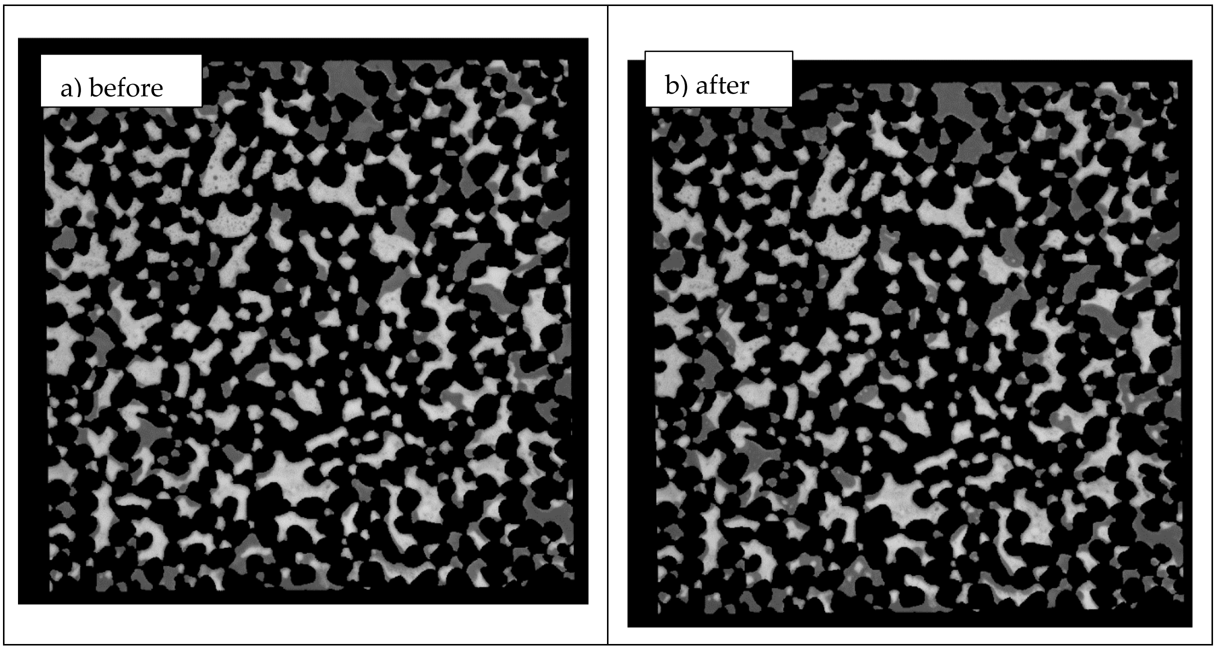

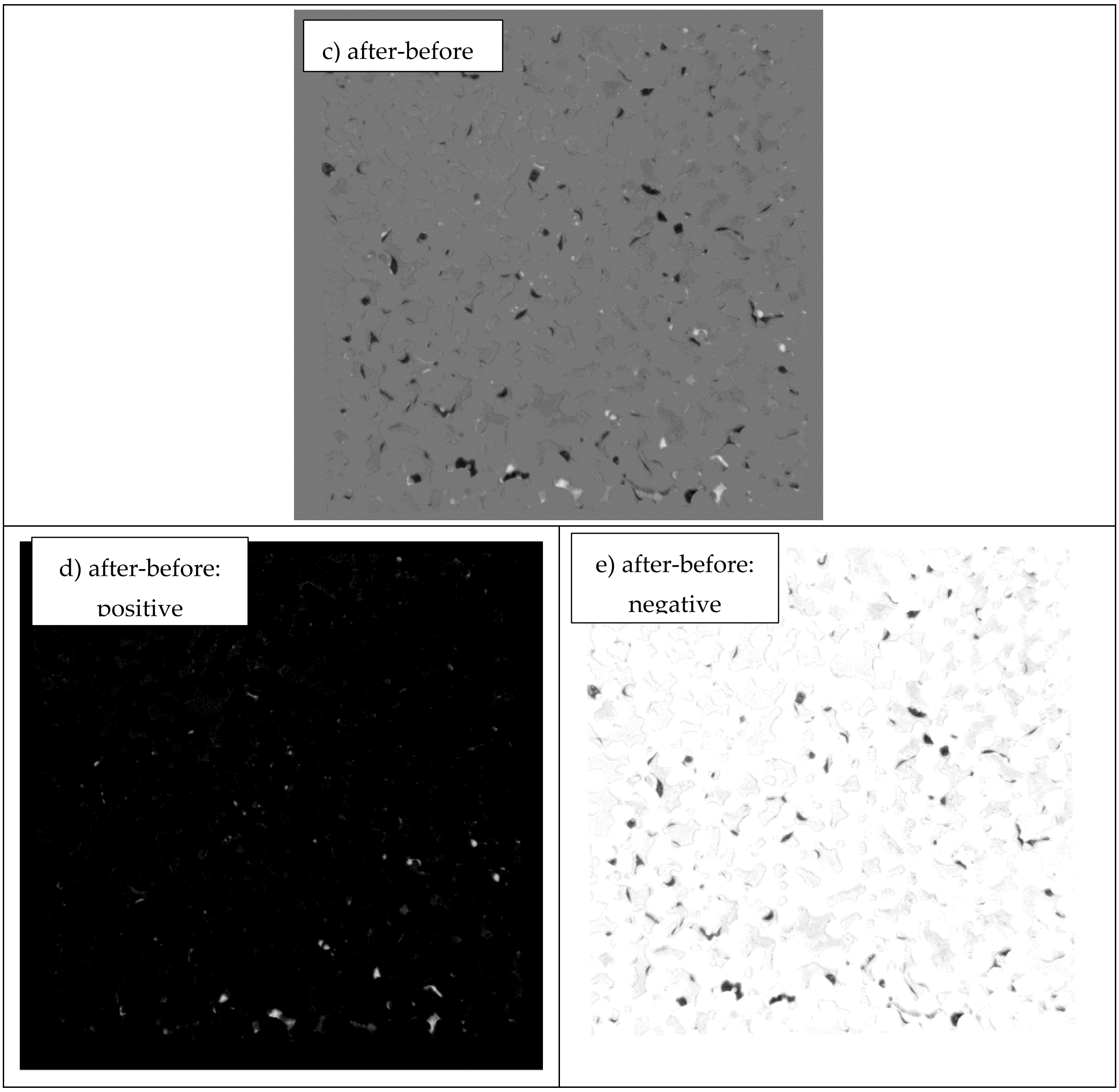

Figure S10. Construction and decomposition of the image difference map shown in Figure 8. Panels a and b show two grayscale images of the deep pore space, taken with some time interval in between. The corresponding intensity patterns are denoted as $I_1(x,y)$ and $I_2(x,y)$. The difference map is defined as $\Delta I(x,y)=I_2(x,y)-I_1(x,y)$. Generally, $\Delta I(x,y)$ has many zero values (indicating the absence of changes) and some clusters of positive and/or negative values. Selecting all pixels where $\Delta I(x,y) \geq 0$ creates $\Delta I^+(x,y)$ while it is $\Delta I^-(x,y)$ for $\Delta I(x,y) \leq 0$: these are defined as the positive and negative difference maps. By definition $\Delta I^+(x,y) + \Delta I^-(x,y) = \Delta I(x,y)$.

To optimally present the difference maps without sacrificing the information that is contained in the grayscale distribution, some intensity offsets or scalings were applied. Since $\Delta I(x,y)$ was defined on an 8-bit scale (0-255) and its extrema were (-116, +114), a constant value of 120 grayscale units was added. This results in the image in panel c): zero changes look gray, negative changes look darker and positive changes look brighter. Generally, negative (/positive) intensity changes mean that brine was displaced by crude oil (/vice versa).

Separately displaying $\Delta I^+(x,y)$ and $\Delta I^-(x,y)$ on an 8-bit grayscale allows to better see the nuances if the full scale is used. Therefore both images were multiplied by 255/116. For $\Delta I^+(x,y)$ this multiplication directly produces panel d). For $\Delta I^-(x,y)$ adding a constant value of 255 is needed to make all pixels positive, while maintaining the trend that darker means more negative. The result is shown in panel e).

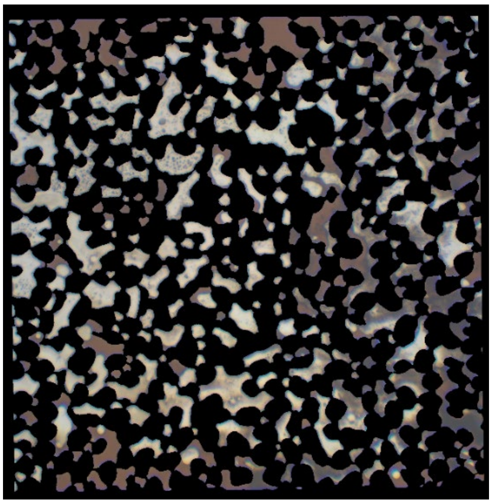

Calcite chip, initial water-flooding at 22°C, ROS=56%(B)-58%(I)
B=binarization method, I=interpolation method

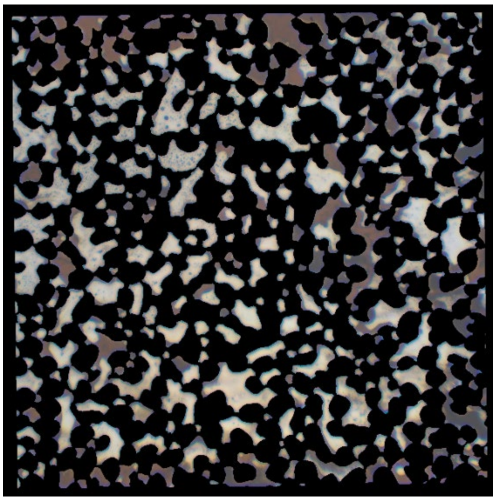

Calcite chip after raising T from 22 to 90°C, ROS=48%(B)-50%(I)

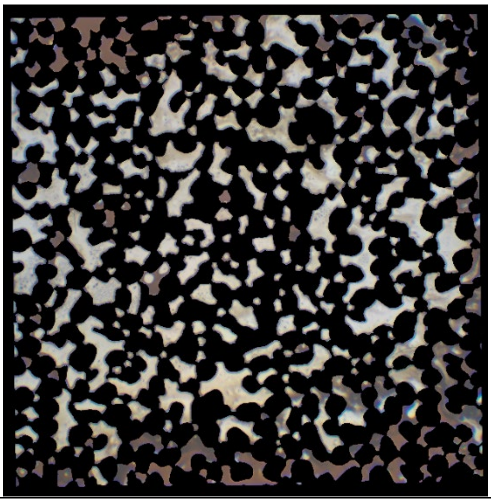

Calcite chip, initial water-flooding at 90°C, ROS=32%(I)-41%(B)

Figure S11. Brine and CRO distributions for the experiments described in Fig 6a and Table 1.

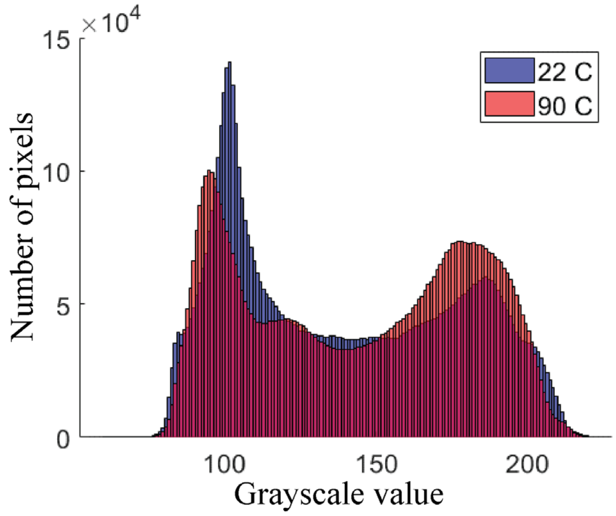

Figure S12. Grayscale intensity histograms (for the deep pores) sampled at different stages of sustained HSW waterflooding of the first experiment: 22 °C (after 3.5 h), and 90 °C (after 48 h). Predominantly, it is the histogram shape that changes with the flooding temperature, i.e. going from 22°C to 90°C the CRO-rich peak (grayscale ~100) gets lower while the main brine-rich peak (grayscale ~180) gets higher. This clearly indicates that CRO has been replaced by brine.

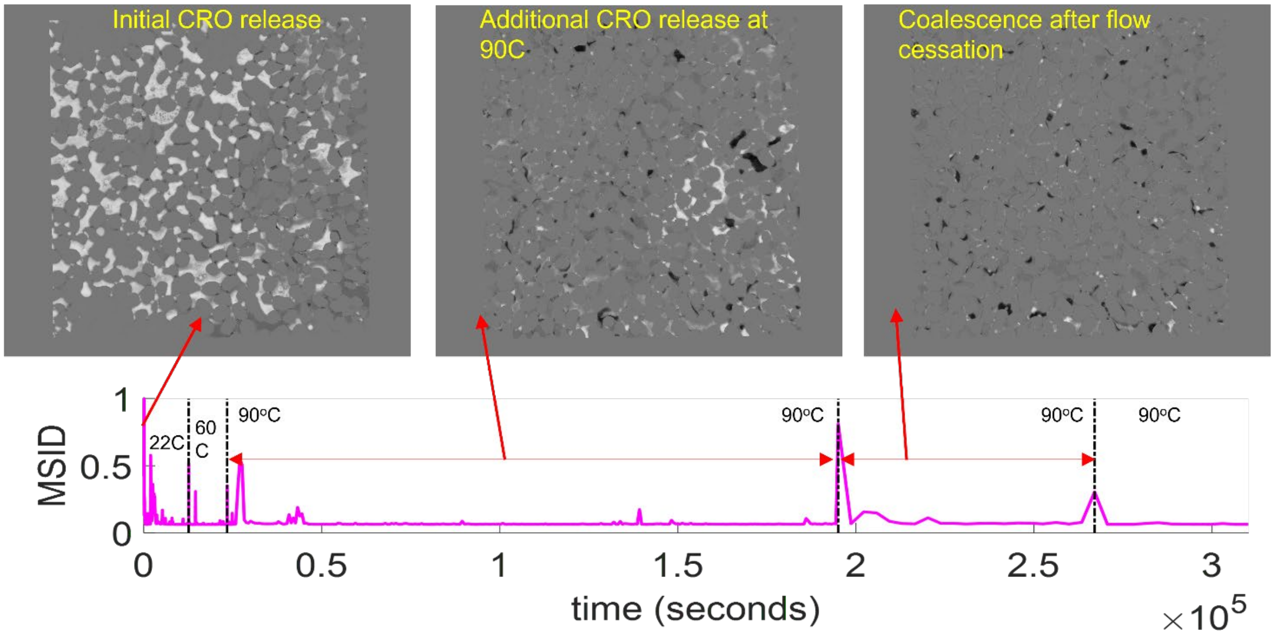

Figure S13. Grayscale difference maps and Mean Squared Intensity Difference signal based on such maps, for the 22-60-90°C waterflooding experiment. The plotted MSID signal is based on differences between 2 subsequent frames, for each pixel the grayscale value difference between frame (i) and frame (i+1) is squared and summed up with the other pixels. A zero MSID value means that the entire grayscale picture did not change between the subsequent frames. The shown grayscale difference maps are obtained by comparing two images that are separated by multiple time frames. For the 'initial CRO release' map, this is a short time, involving a few pore volumes. For the other two maps, the time ranges are indicated by the red arrows in the MSID plot.

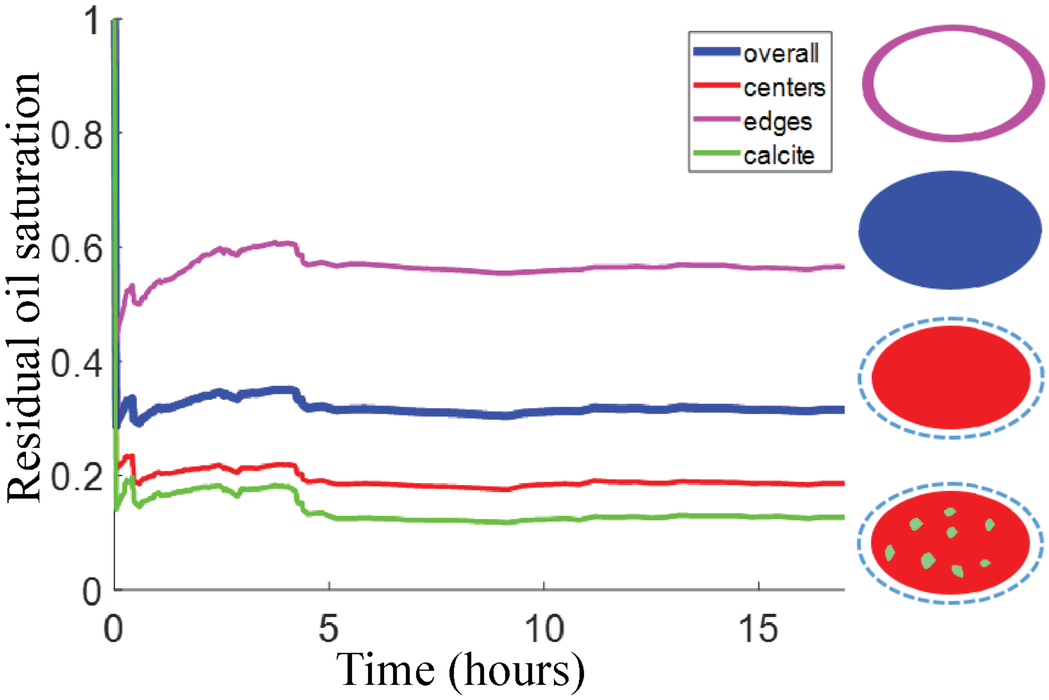

Figure S14. Similar analysis as in Figure 11, for the sample that was waterflooded directly at 90⁰C. The overall ROS (blue) is split into contributions from the centers (red) and the edges (magenta) of the pores. For the pore centers, also the ROS as measured on calcite-coated areas (green) is shown.
